# Sox2 induces glioblastoma cell stemness and tumor propagation by repressing TET2 and deregulating 5hmC and 5mC DNA modifications

**DOI:** 10.1038/s41392-021-00857-0

**Published:** 2022-02-09

**Authors:** Hernando Lopez-Bertoni, Amanda Johnson, Yuan Rui, Bachchu Lal, Sophie Sall, Maureen Malloy, Jonathan B. Coulter, Maria Lugo-Fagundo, Sweta Shudir, Harmon Khela, Christopher Caputo, Jordan J. Green, John Laterra

**Affiliations:** 1 Hugo W. Moser Research Institute at Kennedy Krieger, Baltimore, MD USA; 2 Department of Neurology, Johns Hopkins University School of Medicine, Baltimore, MD USA; 3 Department of Biomedical Engineering, Institute for NanoBioTechnology, and the Translational Tissue Engineering Center, Johns Hopkins University School of Medicine, Baltimore, MD USA; 4 Bloomberg School of Public Health, Department of Environmental Health and Engineering, The Johns Hopkins University School of Medicine, Baltimore, MD USA; 5 The Department of Radiation Oncology and Molecular Radiation Sciences, The Johns Hopkins University School of Medicine, Baltimore, MD USA; 6 Department of Ophthalmology, Johns Hopkins University School of Medicine, Baltimore, MD USA; 7 Department of Oncology, Johns Hopkins University School of Medicine, Baltimore, MD USA; 8 Departments of Materials Science & Engineering and Chemical & Biomolecular Engineering, Johns Hopkins University, Baltimore, MD USA; 9 Department of Neurosurgery, Johns Hopkins University School of Medicine, Baltimore, MD USA; 10 Department of Neuroscience, Johns Hopkins University School of Medicine, Baltimore, MD USA

**Keywords:** Cancer stem cells, Epigenetics

## Abstract

DNA methylation is a reversible process catalyzed by the ten–eleven translocation (TET) family of enzymes (TET1, TET2, TET3) that convert 5-methylcytosine (5mC) to 5-hydroxymethylcytosine (5hmC). Altered patterns of 5hmC and 5mC are widely reported in human cancers and loss of 5hmC correlates with poor prognosis. Understanding the mechanisms leading to 5hmC loss and its role in oncogenesis will advance the development of epigenetic-based therapeutics. We show that TET2 loss associates with glioblastoma (GBM) stem cells and correlates with poor survival of GBM patients. We further identify a SOX2:miR-10b-5p:TET2 axis that represses TET2 expression, represses 5hmC, increases 5mC levels, and induces GBM cell stemness and tumor-propagating potential. In vivo delivery of a miR-10b-5p inhibitor that normalizes TET2 expression and 5hmC levels inhibits tumor growth and prolongs survival of animals bearing pre-established orthotopic GBM xenografts. These findings highlight the importance of TET2 and 5hmC loss in Sox2-driven oncogenesis and their potential for therapeutic targeting.

## Introduction

Cancer is as much an epigenetic disease as it is genetic and we now recognize that DNA methylation, histone modification, chromatin architecture, and RNA-mediated gene regulation play fundamental roles during tumorigenesis.^1^ Alterations in DNA methylation patterns are found in all types of cancer and are thought to drive tumorigenesis.^2^ Hypermethylation of tumor-suppressor genes and de-methylation of oncogenes are strong contributors to tumor initiation, progression and metastasis.^3^ Furthermore, neoplastic cells are thought to become “addicted” to some DNA methylation events.^2^

DNA methylation is established and maintained by the coordinate actions of DNA methyl-transferases (i.e., DNMTs) and DNA de-methylases (e.g., TET1/2/3) and dysregulation of these enzymes is linked to the tumor cell phenotype.^4,5^ DNA methylation generally occurs on cytosine-guanine (CpG) sequences and is established by DNMTs, which catalyze the conversion of cytosine to 5-methylcytosine (5mC). CpG-rich regions or “islands” are associated with gene silencing by recruiting methyl binding proteins that interact with chromatin remodeling enzymes, histone deacetylases, and co-repressors to inhibit gene transcription.^6^ DNA methylation is a reversible process thought to occur spontaneously until the discovery of the ten–eleven translocation (TET) family of enzymes.^7^ These enzymes function as dioxygenases that catalyze the conversion of 5mC to 5-hydroxymethylcytosine (5hmC). Multiple studies identify reduced 5hmC levels in several cancers and correlations between the loss or inactivation of TETs with tumor progression. These associations strongly suggest that TET enzymes activate tumor suppressing mechanisms.^8,9^ However, the mechanisms leading to the loss of 5hmC in cancer and the role this phenomenon plays in establishing and maintaining tumor-propagating cell populations remains poorly understood.

Brain tumors are among the most devastating forms of cancer and glioblastoma (GBM) represents the most aggressive and lethal form of the disease.^10^ GBM is characterized by small subsets of cells, referred to as cancer stem cells (CSCs or glioma stem cells, GSCs) that display stem-like properties.^11^ These GSCs act as critical determinants of GBM resistance to current treatments and play an important role in recurrence.^12^ Notably, alterations in DNA methylation and hydroxyl-methylation patterns have been widely reported in human gliomas and the GSC compartment.^13,14^ In addition, the subtypes of GBM exhibit distinct and abnormal patterns of DNA methylation,^15^ suggesting that DNA methylation plays a central role in the distinct behaviors of GBM subtypes. Multiple studies have found negative correlations between 5hmC levels and glioma grade^16,17^ and loss of 5hmC correlates with poor prognosis of GBM patients.^13,18^ This 5hmC loss is partially explained by isocitrate dehydrogenase (IDH1/2) mutations, which result in the production of 2-hydroxyglutarate (2HG) that inhibits TET enzymatic function.^19^ Interestingly, GBM are also characterized by low levels of 5hmC despite being predominantly IDH wild-type,^20^ suggesting a different mode of TET inactivation in these tumors. Furthermore, reprogramming events contribute to the tumor-propagating phenotype of GBM cells by driving bi-directional transition between stem-like and non-stem-like glioma cells and this plasticity is, in part, governed by changes in DNA methylation.^21,22^ Importantly, targeting this epigenetic dysregulation can impact tumor initiation and propagation in GBM.^21,23^ Yet, the mechanisms leading to 5hmC loss in IDH1/2 wild-type GBM, the downstream effectors of this dysregulation, the role of reprogramming events in this process, and whether these changes can be reversed to achieve therapeutic responses remains to be elucidated.

The goals of this study are to understand the molecular circuits involved in 5hmC dysregulation in GSCs and their contributions to IDH1/2 wild-type GBM oncogenesis. We show for the first time that the stemness-inducing reprogramming transcription factor SOX2 represses the TET2 demethylase and decreases 5hmC, the enzymatic product catalyzed by TET proteins, in IDH1/2 wild-type GSCs. Patient-derived IDH1/2 wild-type GSC self-renewal and capacity to grow as tumor xenografts are shown to be enhanced by shRNA hairpins that repress TET2 and 5hmc levels. We show that the miR-10b-5p onco-miR is induced by SOX2, directly targets TET2, mediates onco-methylation, GSC induction and glioma malignancy. In vivo delivery of miR-10b-5p antagomirs to pre-established orthotopic GBM xenografts using advanced poly(beta-amino ester) (PBAE) polymers reduced tumor growth and prolonged animal survival. These findings establish a mechanism by which SOX2 drives IDH1/2 wild-type GBM stemness and oncogenesis by altering DNA methylation and hydroxymethylation. We establish proof-of-concept supporting the therapeutic efficacy of targeting this novel mechanism of oncogenic epigenetic dysregulation.

## Results

### TET2 expression and function in clinical GBM and patient-derived GSCs

**Fig. 1:**
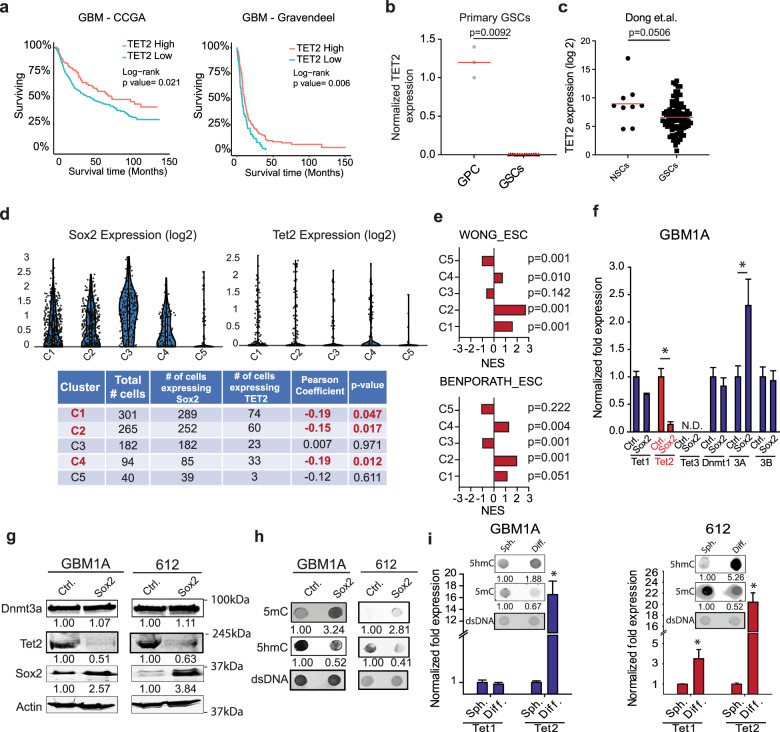
SOX2 decreases TET2 expression and 5hmC levels in GSCs and correlates with loss of TET2 in GBM. **a** Kaplan–Meier survival curves comparing GBM patients across multiple datasets. Survival data were retrieved from the GlioVis portal (http://gliovis.bioinfo.cnio.es). We analyzed datasets that included more than 100 patients. Survival analysis was restricted to patients with primary, IDH1/2 wild-type GBM. **b** qRT-PCR analysis showing decreased TET2 mRNA in low-passage primary GSC isolates. **c** RNA-Seq data comparing TET2 expression in neural stem cells (NSCs) and glioma stem cells (GSCs). **d** Violin plot showing the expression of SOX2 and TET2 across five cell clusters. The mRNA level is shown on *y*-axis as log2 expression and the *x*-axis represents the different clusters. scRNA-Seq expression data were retrieved and analyzed with BBrowser (v 2.44.4). **e** Normalized enrichment scores (NES) from gene-set enrichment analysis showing clusters 1, 2, and 4 are enriched for embryonic stem cell (ESCs) signatures. **f** qRT-PCR analysis showing the selective decrease in TET2 mRNA and increase in DNMT3A mRNA in GSCs expressing exogenous SOX2. **g** Western blots showing decreased TET2 protein and no change in DNMT3A protein after transgenic SOX2 expression. **h** Dot blot assay showing global increase in 5mC and reduced 5hmC after transgenic SOX2 expression in GSCs. **i** qRT-PCR analysis showing increased TET2 mRNA and no change in TET1 mRNA following forced differentiation of GSCs. Dot blot analysis of genomic DNA isolated from GSCs showing increased 5hmC and decreased 5mC after forced differentiation (inset). Statistical significance was calculated using Student’s *t-*test **b**, **c**, **f**, and **i** and data are presented as mean ± SD. *p < 0.05

To explore the potential clinical impact of altered 5hmC levels in GBM, we examined the relationships between TET expression and patient outcomes and found across multiple clinical datasets that low TET2 expression correlates with poor survival (Fig. 1a and Supplementary Fig. 1). Interestingly and seemingly contradictory, we observed either no significant difference or a modest increase in Tet2 mRNA from bulk tumor tissues compared to non-tumor in the same datasets (Supplementary Fig. 2). GBM is highly heterogeneous at the cellular level.^18^ Tumor-propagating stem-like cell subsets have a dominant role in outcomes and analysis of bulk tumor expression data may mask gene expression correlations in critical cell subpopulations. Quantitative RT-PCR analysis of a panel of low-passage patient-derived primary GBM neurospheres enriched for GSCs revealed very low TET2 expression relative to non-neoplastic glial progenitor cells (GPCs) (Fig. 1). A similar trend in TET expression was found between non-neoplastic neural stem cells and GSCs in available RNA-Seq datasets^24^ (Fig. 1c and Supplementary Fig. 3). To further explore TET2 expression in GBM cell subsets, we analyzed scRNA-Seq expression data from GBM clinical samples using the BioTouring browser.^25^ This analysis revealed that only a small fraction of cells expressing SOX2, a transcriptional driver of GBM stemness, expressed TET2 (Fig. 1d). In addition, we found a significant negative correlation between SOX2 and TET2 expression in three of the five cell subpopulations identified (Fig. 1d, bottom panel). Furthermore, the clusters showing a negative correlation between SOX2 and TET2 expression were enriched with embryonic stem cell signatures (Fig. 1e), suggesting that high expression of SOX2 and low expression of TET2 cooperate to support stem-like cell subpopulations in GBM.

### Loss of TET2 enhances GBM cell stemness and induces a more aggressive tumor phenotype

As mentioned earlier, changes in cell fate mediated by reprogramming transcription factors are accompanied by extensive epigenetic remodeling.^26^ We recently published that the combined action of reprogramming transcription factors OCT4 and SOX2 drive the stem cell phenotype of GBM cells by inducing DNMTs and modifying DNA methylation.^21^ DNA de-methylation is an active process that plays a major role in establishing and maintaining the methylome and the TET family of proteins have a major role in this process.^27^ To dissect if TETs contribute to the hypermethylation induced by OCT4 and/or SOX2, we expressed OCT4 or SOX2 in GBM neurospheres using lentiviral vectors and measured expression of DNMTs (Dnmt1/3A/3B) and TETs (Tet1/2/3) using qRT-PCR. While OCT4 significantly increased DNMT1 and DNMT3A mRNA expression (Supplementary Fig. 4), consistent with our previous findings,^21^ we found a robust reduction of TET2 mRNA and protein in response to SOX2 forced expression (Fig. 1f). Interestingly, despite a modest but significant increase in Dnmt3A mRNA, we did not measure a significant increase in DNMT3A protein after the expression of exogenous SOX2 (Fig. 1g). We could not detect TET3 gene expression in these cells using 2 different sets of primers. Furthermore, the reduction in TET2 observed after transgenic SOX2 expression was sufficient to induce hypermethylation in GSCs as measured by a global increase in 5mC and a global decrease in 5hmC (Fig. 1h). Moreover, we observed a robust increase in TET2 gene expression following forced differentiation of GBM spheres (Fig. 1i and Supplementary Fig 5), conditions that also result in substantial reductions in global DNA methylation (inset, Fig. 1i and Supplementary Fig 5). Taken together, these results show that SOX2 modifies the methylome of GBM cells, at least in part, by repressing TET2 expression and TET2-mediated conversion of 5mC to 5hmC.

**Fig. 2:**
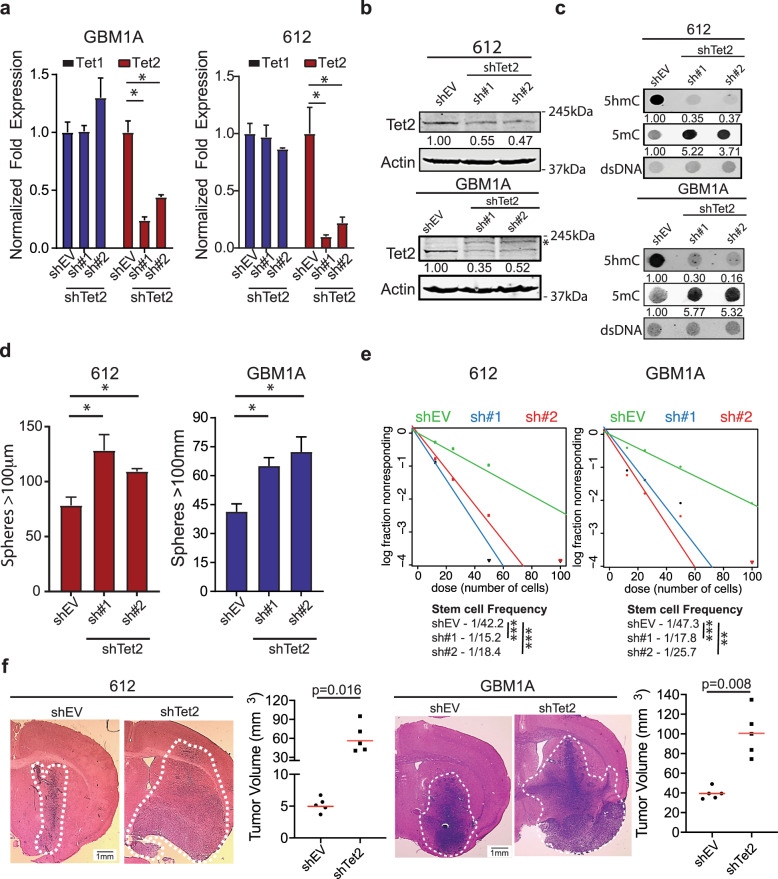
TET2 inhibition enhances the stem and tumor phenotype of GBM cells. **a** qRT-PCR shows specific knockdown of TET2 expression after transduction of shTET2 constructs in patient-derived GSC isolates. Western blot (**b**) and dot blot (**c**) showing shRNA-mediated inhibition of TET2 reduces TET2 protein, reduces 5hmC, and increases 5mC in GSC isolates. * denotes non-specific band. **d** Equal numbers of GSC isolates transduced with lentiviral constructs expressing two distinct shRNAs targeting TET2 or a control vector (shEV) were cultured in a neurosphere medium for 14 days. Quantification of neurospheres (>100 µm diameter) by computer-assisted image analysis shows that TET2 knockdown enhances neurosphere formation. **e** Limiting dilution assay (LDA) in GSC isolates transduced with a control lentivirus or a lentivirus expressing two distinct shRNA hairpins against TET2. **f** Mice were implanted with equal numbers of GSCs transduced with lentiviral constructs expressing an shRNA targeting TET2 (*N* = 5) or a control vector (shEV; *N* = 5). Brains from animals sacrificed 35 (612) or 60 (GBM1A) days after cell implantation show a marked increase in invasive tumor growth. Tumor volumes were calculated from maximum tumor cross-sectional areas determined from H&E-stained sections. One-way ANOVA with Tuckey’s post hoc test was used to calculate statistical significance in **a** and **d**. Statistical significance was calculated using Student’s *t*-test in **f**. Data are presented as mean ± SD. *p < 0.05

Our results suggest that TET2 suppresses GBM cell stemness and malignancy and predict that loss of TET2 with consequent diminished 5hmC will enhance GBM cell stemness and tumor aggressiveness. To test this hypothesis, we knocked down TET2 mRNA expression using two independent shRNA hairpins. An empty vector (shEV) was used as a negative control. TET2 knockdown efficiently decreased TET2 mRNA and protein without affecting TET1 expression (Fig. 2a, b) in GBM neurospheres. Loss of TET2 concurrently reduced global 5hmC levels, increased global 5mC levels (Fig. 2c) and increased GBM self-renewal as neurospheres and GSC frequency in multiple GSC isolates (Fig. 2d, e and Supplementary Figs. 6 and 7). GSC frequency and self-renewal capacity predict tumor growth capacity in vivo.^28^ To examine if TET2 inhibition affects the growth of GBM xenografts, the neurosphere lines described above were implanted to caudate-putamen of immune-deficient mice and all animals were sacrificed when the group implanted with TET2 knockdown cells began to show adverse signs of tumor burden. Histopathological examination showed significantly larger and more invasive tumors in response to TET2 inhibition in two distinct cell models (Fig. 2f). Taken together, these clinical and molecular data show that TET2 downregulation by SOX2 drives GBM cell stemness and in vivo tumor growth.

### The Sox2:miR-10b:TET2 axis modifies the DNA methylation landscape in GSCs

**Fig. 3:**
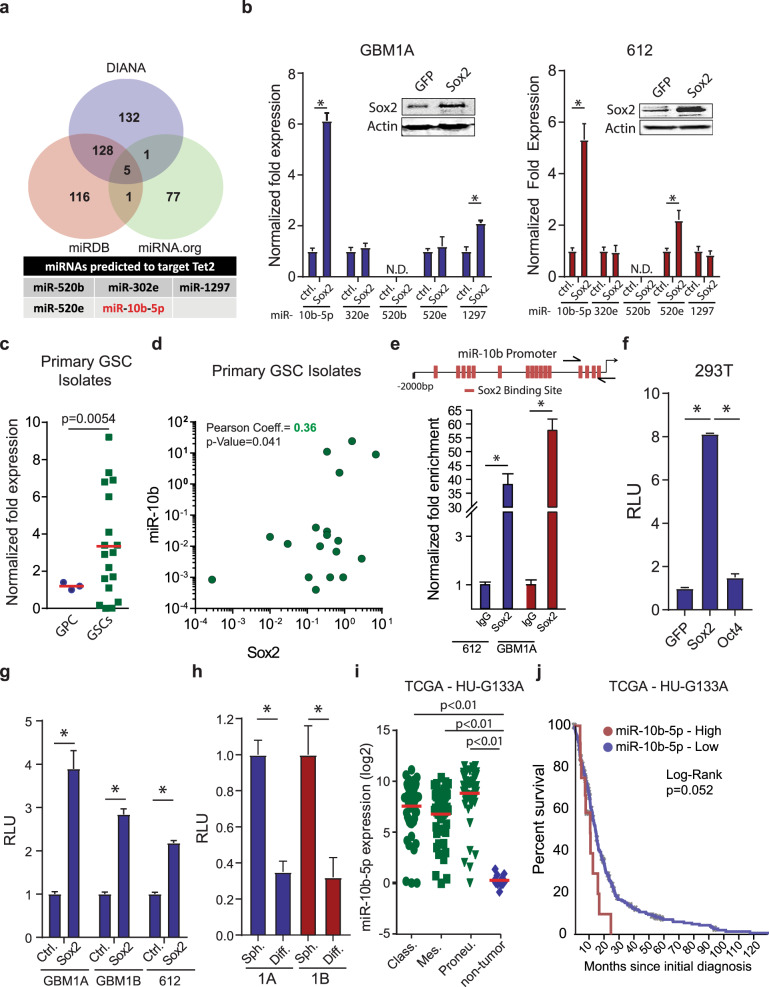
miR-10-5p is activated by SOX2 and correlates with the stem cell and tumor phenotype of GBM. **a** Venn diagram showing the intersection of TET2-targeting miRNAs predicted using three different algorithms (top panel). List of high-confidence miRNAs predicted to target TET2 (bottom panel). **b** qRT-PCR to measure the expression of pre-cursor miRNAs predicted to inhibit TET2 in GSCs expressing transgenic SOX2. Western blot showing SOX2 protein levels in GSCs expressing transgenic Sox2 (inset). **c** qRT-PCR to measure miR-10b-5p expression in GBM neurospheres lines and primary GBM neurosphere isolates compared to non-tumorigenic glial progenitors (normal). **d** Correlation between Sox2 and miR-10b-5p expression in primary GSC isolates. **e** Sox2 binding sites on the human miR-10b-5p promoter, arrows indicate primer sites used for PCR analyses (top panel). DNA purified from chromatin immunoprecipitation was analyzed by qRT-PCR using primer pairs designed to amplify fragments containing Sox2 (bottom panels). **f** 293T cells were co-transfected with a luciferase reporter construct spanning the miR-10b-5p putative promoter containing the SOX2 binding sites and GFP, OCT4, or SOX2 and luciferase activity was measured 2 days after transfection. **g** GSC isolates expressing exogenous SOX2 were transfected with the luciferase reporter construct spanning the miR-10b-5p putative promoter containing the SOX2 binding sites and luciferase activity was measured 3 days after transfection. **h** GBM1A and GBM1B neurospheres were transfected with luciferase reporter construct covering the miR-10b-5p putative promoter containing the SOX2 binding sites and forced to differentiate. Luciferase activity was measured 3 days after differentiation. **i** miR-10b-5p levels in normal brain compared to GBM subtypes. **j** Kaplan–Meier survival curves comparing GBM patient survival based on miR-10b-5p expression. miR-10b-5p expression, and patient survival data were retrieved from the TCGA database using the BETASTASIS portal (http://www.betastasis.com). One-way ANOVA with Tuckey’s post hoc test was used to calculate statistical significance in **f** and **i**. Statistical significance was calculated using Student’s *t*-test in **b**, **c**, **d**, **f**, **g**, and **h**. Data are presented as mean ± SD. *p < 0.05

MicroRNAs regulate multiple biological processes including epigenetics and tumorigenesis^29^ and have been found to contribute to TET2 dysregulation and hematopoietic stem cell transformation.^30^ We have reported that OCT4 and SOX2 regulate a focused panel of miRNAs that impact GBM cell stemness and malignancy via multiple mechanisms involving DNMTs and regulators of chromatin architecture.^21,31^ Using these previously validated approaches for identifying GSC-regulating miRNAs, we asked if SOX2 activates onco-miRs that repress TET2 expression and thereby regulate GSCs. Set-distribution analysis of DIANA, miRDB, and miRNA.org prediction algorithms identified five high-confidence candidate miRNAs (Fig. 3a). MiR-10b-5p was the only one of these five candidate TET2-targeting miRNAs that was consistently induced by Sox2 in multiple patient-derived GSC lines tested (Fig. 3b and Supplementary Fig. 8). In addition, miR-10b-5p was the only miRNA in this subset to be repressed by forced GSC differentiation, a condition that strongly inhibits SOX2 expression^21^ and induces TET2 expression (Supplementary Figs. 9 and 1i). The functional link between miR-10b-5p and SOX2 in GSCs was supported further by high levels of miR-10b-5p expression in low-passage GSC isolates compared with GPCs (Fig. 3c) and the significant positive correlation between SOX2 and miR-10b-5p expression in GSCs (Fig. 3d). Consistent with these molecular associations, bioinformatics analysis of the miR-10b-5p promoter identified multiple SOX2 binding sites (and no OCT4 sites as control) within 2 kb of the miR-10b-5p transcription start site (Fig. 3e, top and Supplementary Fig. 10). ChIP-PCR analysis of a subset of putative binding sites confirmed SOX2 binding to the miR-10b-5p promoter in two distinct GSC isolates (Fig. 3e, bottom), supporting the potential for SOX2 to directly regulate miR-10b-5p expression in GSCs. Exogenous SOX2, but not GFP or OCT4 as controls, induced luciferase expression from a reporter containing the respective miR-10b-5p promoter transcription factor binding sites in 293T cells (Fig. 3f) and in multiple GBM neurospheres stably expressing transgenic SOX2 (Fig. 3g). Conversely, luciferase expression was inhibited in neurospheres following their forced differentiation, consistent with repression of endogenous SOX2 and its regulation of miR-10b-5p under “physiologic” conditions (Fig. 3h). The clinical relevance of these findings is supported by miR-10b-5p upregulation in GBM clinical specimens compared to non-neoplastic tissue (Fig. 3i) with high miR-10b-5p expression correlating with poor patient outcome (Fig. 3j).

**Fig. 4:**
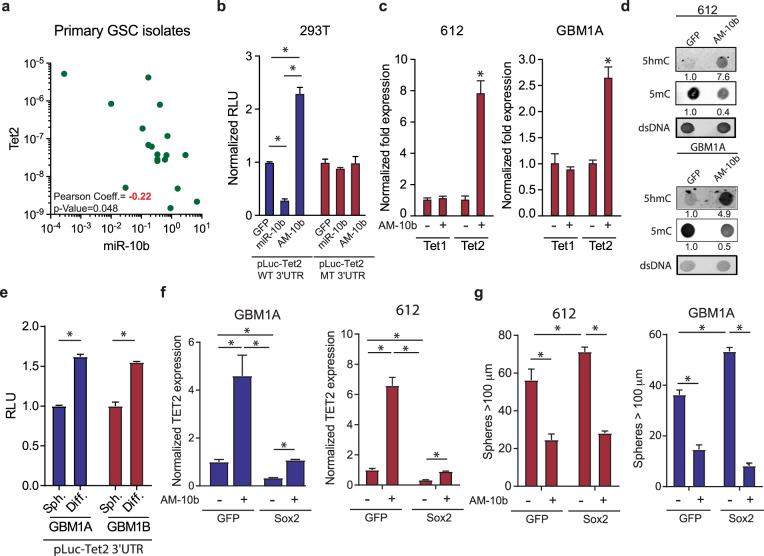
miR-10-5p is a direct regulator of TET2 and 5hmC expression in GBM cells. **a** Correlation between TET2 and miR-10b-5p expression in primary GSC isolates. **b** 293T cells were co-transfected with a luciferase reporter construct spanning the TET2 3′UTR containing either wild-type (WT 3′UTR) or mutated (MT 3′UTR) miR-10b-5p binding sites and a plasmid expressing a control miRNA (GFP), a miR-10b-5p inhibitor (AM-10b-5p), or a miR-10b-5p mimic. **c** qRT-PCR analysis showing increased TET2 mRNA and no change in TET1 mRNA following miR-10b-5p inhibition in GSCs. **d** Dot blot analysis of genomic DNA isolated from GSCs transduced with a lentiviral vector expressing the miR-10b-5p inhibitor (AM-10b-5p) showing increased 5hmC and decreased 5mC. **e** GBM1A and GBM1B neurospheres were transfected with luciferase reporter construct covering the TET2 3′UTR containing the miR-10b-5p binding site and forced to differentiate. Luciferase activity was measured 3 days after differentiation. **f** qRT-PCR analysis measuring expression of TET2 in GSC isolates transduced with SOX2 ± a miR-10b-5p inhibitor (AM-10b-5p). **g** Equal numbers of GSC isolates transduced with SOX2 ± a miR-10b-5p inhibitor (AM-10b-5p) were cultured in neurosphere medium for 14 days. Quantification of neurospheres (>100 µm diameter) by computer-assisted image analysis shows that miR-10b-5p inhibits neurosphere formation independent of transgenic Sox2 expression. One-way ANOVA with Tuckey’s post hoc test was used to calculate statistical significance in **b**, **f**, and **g**. Statistical significance was calculated using Student’s *t*-test in **c** and **e**. Data are presented as mean ± SD. *p < 0.05

Tet2 and miR-10p-5p expression levels were found to be inversely correlated in primary GSC isolates (Fig. 4a). Direct TET2 targeting by miR-10b-5p is supported by bioinformatics analysis showing that the seed region for miR-10b-5p is highly conserved among several species in the TET2 3′UTR (Supplementary Fig. 11a). Luciferase-reporter assays were used to determine if miR-10b-5p directly binds this region of the TET2 3′UTR. The human TET2 3′UTR containing either wild-type or the mutated miR-10b-5p binding site was cloned into a luciferase reporter cassette (pLuc-Tet2 WT 3′UTR or pLuc-TET2 MT 3′UTR) and co-transfected into HEK293T in the presence of a scrambled miRNA vector expressing GFP, a miR-10b-5p inhibitor (AM-10b-5p), or miR-10b-5p mimic. Compared to cells transfected with the control vector, miR-10b-5p inhibition induced luciferase activity ~2-fold and transgenic miR-10b-5p expression reduced luciferase activity by ~70% in cells transfected with pLuc-TET2 WT 3′UTR and had no effect in cells transfected with pLuc-TET2 MT 3′UTR (Fig. 4b). Inhibition of miR-10b-5p using an antagomir (AM-10b-5p) in GBM neurospheres increased TET2 mRNA without affecting TET1 gene expression (Fig. 4c and Supplementary Fig. 11b) and concurrently increased 5hmC and decreased 5mC levels (Fig. 4d). In addition, conditions of neurosphere forced differentiation shown to increase TET2 expression and 5hmC levels (Fig. 1i) repressed miR-10b-5p expression as evidenced by increased pLuc-TET2-3′UTR transgene expression (Fig. 4e). Furthermore, inhibiting miR-10b-5p with AM-10b-5p inhibited the capacity of SOX2 to repress TET2 expression (Fig. 4f) and prevented the induction of neurosphere formation by SOX2 (Fig. 4g). Taken together these results demonstrate that miR-10b-5p functions to mediate TET2 inhibition and GBM neurosphere formation by SOX2.

### Suppression of miR-10b-5p inhibits tumor growth and prolongs animal survival in an orthotopic model of human GBM

**Fig. 5:**
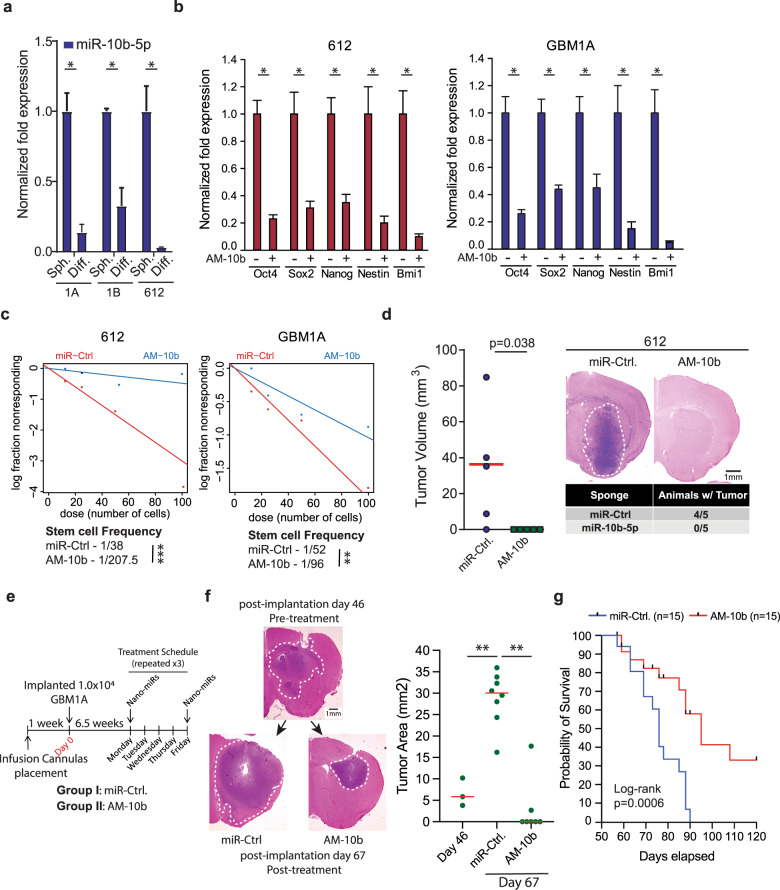
miR-10b-5p inhibition represses the stem cell and tumor phenotype of GBM cells. **a** qRT-PCR analysis shows a decreased expression of pre-miR-10b-5p 5 days after forced differentiation of GBM neurospheres. **b** qRT-PCR showing expression of stem cell markers and drivers 4 days after miR-10b-5p inhibition. **c** Limiting dilution assay (LDA) in GSC isolates transduced with a control lentivirus or a lentivirus expressing a miR-10b-5p inhibitor (AM-10b-5p). **d** Mice were implanted with equal numbers of viable GSCs transduced with lentiviral constructs expressing a miR-10b-5p inhibitor (*N* = 5) or a control vector (miR-Ctrl.; *N* = 5). Brains from animals sacrificed 42 days after cell implantation show a marked decrease in tumor growth. Tumor volumes were calculated from maximum tumor cross-sectional areas determined from H&E-stained sections. **e** Schematic summarizing the treatment schedule for in vivo delivery of nano-miRs. **f** Representative H&E-stained brain sections from mice implanted with GBM1A neurosphere cells treated with a control nano-miR (*n* = 8) or the miR-10b-5- inhibitor (AM-10b) (*n* = 7). Animals were sacrificed 67 days after cell implantation. Maximum tumor cross-sectional areas following treatment with nano-miRs representing viable tumor tissue (right panel). **g** Kaplan–Meier survival curves comparing mice treated with control nano-miRs (miR-Ctrl) or miR-10b-5p inhibitor (AM-10b). Therapy in the survival study was initiated 45 days after tumor cell implantation. Survival was compared across arms using the log-rank test (*N* = 15). Statistical significance was calculated using Student’s *t*-test in **a**, **b**, and **d**. One-way ANOVA with Tuckey’s post hoc test was used to calculate statistical significance in **f**. Data are presented as mean ± SD. **p < 0.01; *p < 0.05

We showed that the SOX2 reprogramming TF that drives GBM cell stemness and tumor-propagating potential induces miR-10b-5p (Fig. 3) that targets TET2 (Fig. 4 and Supplementary Fig. 12) that we show inhibits GBM cell stemness and GBM xenograft growth (Fig. 2). These findings predict that miR-10b-5p is required to maintain the tumor-propagating stem-like phenotype of GBM cells. Consistent with this hypothesis, forced differentiation of GBM neurospheres decreased levels of pre-cursor (pre-) miR-10b-5p in multiple neurosphere lines (Fig. 5a). Inhibiting endogenous miR-10b-5p in GBM neurospheres inhibited the expression of drivers and markers of the stem cell phenotype (Fig. 5b) and reduced their stem cell frequency and capacity to self-renew as spheres (Fig. 5c). To investigate the effects of miR-10b-5p inhibition on tumor-propagating capacity in vivo, low-passage GSC isolates stably transduced in vitro with the AM-10b-5p or a control miRNA were implanted to the putamen of nude mice (5 mice/group). Histopathological examination of brains from animals sacrificed 42 days post cell implantation showed that AM-10b-5p drastically inhibited tumor-initiation capacity of GSCs in vivo (Fig. 5d).

The capacity of in vivo miR-10b-5p inhibition to treat orthotopic GBM and prolong animal survival was evaluated using a clinically translatable miRNA delivery platform^23,32^ consisting of the miR-10b-5p antagomiR (or control scrambled miRNA) encapsulated in biodegradable PBAE-nanoparticles. PBAE-nanoparticle-based delivery of AM-10b-5p nano-miRs to GBM neurospheres in vitro significantly decreased levels of endogenous miR-10b-5p by ~70%, significantly increased TET2 mRNA and 5hmC levels, and inhibited GBM neurosphere cell growth and self-renewal as spheres without affecting cell viability, recapitulating results using the lentiviral based AM-10b-5p delivery (Supplementary Fig. 13). Having established the effectiveness of nanoparticle-based AM-10b-5p in vitro, mice bearing large pre-established orthotopic patient-derived GBM xenografts (46 days post tumor cell implantation) were treated with either control or AM-10b-5p nano-miRs by direct intra-tumoral infusion twice per week for 3 weeks (Fig. 5e), using our established methods.^23,32^ Tumors treated with AM-10b-5p nano-miRs regressed compared to the growth of tumors treated with control nano-miRs (*p* < 0.01) (Fig. 5f). The capacity for AM-10b-5p to prolong the survival of animals bearing orthotopic GBM xenografts was examined. Orthotopic GBM xenografts were established and nano-miR therapy was administered beginning on post-tumor cell implantation day 45 as described above. Mice bearing pre-established orthotopic patient-derived GBM xenografts were treated with either control or AM-10b-5p nano-miRs by direct intra-tumoral infusion twice per week for 3 weeks and animals (*n* = 15 in each group) were then monitored without further therapy for survival. Median survival for animals treated with control nano-miRs was 72 days and all control-treated animals were either dead or pre-morbid requiring euthanasia by post-implantation day 90. Median survival of animals treated with AM-10b-5p nano-miRs was 97 days and 5 of 15 animals remained alive with normal behavior at post-implantation day 120, at which time the experiment was terminated (Fig. 5g). These results show that inhibiting the Sox2:miR-10b-5p:TET2 axis inhibits GBM growth and prolongs the survival of animals bearing large orthotopic GBM.

## Discussion

Epigenetic modes of gene regulation are essential to maintaining proper control of physiological transcriptional programs and methylome alterations result in disease, including cancer. Low levels of 5hmC are observed in several cancers and loss of 5hmC is widely viewed as an epigenomic hallmark of GBM.^9,18^ Histopathology studies show that brain tumor cells express lower 5hmC compared to normal brain^13^ and there is a negative correlation between 5hmC levels and glioma grade, with grade IV brain tumors having very low levels of this DNA modification.^13,33^ These observations are partially explained by isocitrate dehydrogenase (IDH1/2) mutations, which result in the production of 2HG that inhibits TET enzymatic function.^19^ Interestingly, GBM are also characterized by low levels of 5hmC despite being predominantly IDH wild-type,^20^ suggesting a different mode of TET inactivation in these tumors. Genomic analysis of a small group of GBM patient samples suggests that the TET2 loci is prone to hypermethylation leading to repression, providing an alternative explanation for the robust loss of 5hmC observed in GBM.^34^ We now show, for the first time, that forced expression of reprogramming transcription factor SOX2, which is highly expressed in GBM, reduces expression of TET2 and 5hmC (Fig. 1f, g), thus contributing to the hyper-methylated phenotype of GSCs (Fig. 1h).

The function of miRNAs is closely linked to the coding-genes they regulate making their mechanism of action and biological effects extremely context specific.^35^ Despite miR-10b-5p being reported as an oncogene in multiple cancers,^36–39^ it also has tumor-suppressor properties in certain contexts including clear-cell renal cell carcinoma,^40^ gastric cancer,^41^ and cervical cancer.^42^ In addition, miR-10b-5p can enhance the stem cell phenotype in multiple contexts. For instance, miR-10b-5p has been shown to support self-renewal of breast CSCs by modulating the PTEN/AKT pathway^37^ and can drive the stem cell phenotype of cutaneous squamous cell carcinomas.^43^ We identified miR-10b-5p as a direct target of SOX2 (Fig. 3) and demonstrate this miRNA regulates TET2 and 5hmC levels in GSCs (Fig. 4 and Supplementary Fig. 6). This miRNA is upregulated in GSC primary isolates compared to GPCs (Fig. 3c) and over-represented in GBM compared to non-tumor samples (Fig. 3i). In addition, high miR-10b-5p expression correlates with poor patient outcome in GBM (Fig. 3j) and transgenic expression of miR-10b-5p enhances the stem cell phenotype of GBM cells (Supplementary Fig. 12). In GBM, miR-10b-5p can regulate cell cycle through MBNL1-3, SART3, RSRC1 in GBM^44^ stem-like cells; however, its relationship to reprogramming events and 5hmC regulation was unknown. Our findings are consistent with research showing that miR-10b-5p expression is a strong marker of poor prognosis for GBM and can regulate the tumor and stem cell phenotype of GBM cells.^39,45,46^ These results demonstrate that the SOX2:miR-10b-5p:TET2 axis acts as a critical mediator of onco-methylation in GSCs and highlights a new putative mechanism of 5hmC reduction in GBM.

Loss or inactivation of TET enzymes and deregulation of 5hmC are emerging as critical determinants of the CSC and tumor phenotypes.^47,48^ Despite TET2 not being often mutated in gliomas,^17^ recent studies show frequent TET2 downregulation.^49^ Chen et al. show that there is a negative correlation between TET2 expression and glioma grade and provide evidence using non-stem-like cell GBM models that this repression is mediated by Zinc finger E‑box‑binding homeobox 1 (ZEB1),^49^ indicating that loss of TET2 regulates oncogenic event in gliomas. Loss or inactivation of TET2 enhances self-renewal capacity of hematopoietic stem cells and induces myeloid transformation and leukemogenesis.^30,50^ Analysis of the global distribution of 5hmC in clinical GBM specimens identified new patterns of aberrant DNA hypermethylation thought to regulate gliomagenesis, in part, by affecting transcriptional programs involved in regulating stem cell maintenance.^51^ Consistent with these observations, different GSC subtypes also display different methylation patterns;^52^ however, how TET2-driven changes in 5hmC affect this molecular phenotype remains unknown. Ectopic expression of TET2 impairs tumor growth capacity of GBM cells and this phenotype is associated with activation of neural differentiation programs.^34,49^ These findings suggest that TET2 and 5hmC regulate the tumor phenotype of GBM by controlling the stem cell phenotype of GBM cells. Consistent with these clinical and bioinformatics predictions, our results show that TET2 expression is reduced in primary GSC isolates compared to GPCs (Fig. 1b, c) and low TET2 expression correlates with poor patient outcomes in GBM (Fig. 1a). We also show that TET2 knockdown using two independent shRNA hairpins efficiently decreases 5hmc levels and significantly enhances self-renewal and tumor growth capacity of GSC isolates (Fig. 2 and Supplementary Fig. 6). These results show, for the first time, that loss of TET2 directly affects the stem cell and tumor phenotype of GBM cells and predict that strategies focused on re-expressing TETs and/or normalizing 5hmC levels can be developed as anti-cancer molecular therapeutics.

As mentioned earlier, loss of 5hmC is observed in multiple cancers and approaches focused on normalizing 5hmC expression in tumors show promise as modes to inhibit the CSC phenotype and impede tumor growth.^53^ For instance, TET2 restoration inhibits self-renewal and tumor-propagating capacity of leukemia cells by inducing de-methylation.^54^ Similarly, the rescue of 5hmC loss by expressing transgenic TET2 in ovarian cancer cells reduced cell stemness and restored sensitivity to chemotherapy in vitro and in vivo.^55^ In GBM, expression of transgenic TET2 activates neural differentiation programs and inhibits tumor growth capacity of non-stem-like GBM cells.^34^ Likewise, TET3 expression has been reported to inhibit GSC self-renewal and tumorigenesis.^56^

Despite these exciting results, developing therapeutic approaches based on TET re-expression may prove challenging due to the size of these enzymes. Therefore, finding alternative ways to activate TETs or increase their expression in tumor cells may prove more efficacious. RNAi-mediated mechanisms of cell fate regulation combined with nanomedicine are gaining traction as avenues to develop innovative molecular therapeutics. Thus far, three siRNA-based therapeutics have been approved by the Food and Drug Administration, Patisiran, Givosiran, and Lumasiran^57,58^ with multiple others currently in clinical trials.^59^ These promising developments open the door for new therapeutic approaches based on reconstituting tumor-suppressive miRNAs or inhibiting oncogenic miRNAs as means to normalize dysregulated molecular networks, inhibit tumor growth, and enhance the effects of current standards-of-care.^23,32,60^ Emerging evidence shows that non-coding RNAs play a critical role in controlling the expression of TET2 in GBM cells.^61^ Ren et al. demonstrate that long non-coding RNA AC016405.3 regulates proliferation and migration of non-stem-like GBM cells by sponging miR-19a-5p and therefore modulating TET2 expression,^61^ suggesting that utilizing non-coding RNAs to modulate TET2 expression can impact GBM growth. Our results show that in vivo delivery of miR-10b-5p antagomirs using advanced PBAE polymers reduces tumor growth and prolongs survival in orthotopic GBM xenograft models (Fig. 5f, g), highlighting the potential of miR-10-5p inhibition as an impactful anti-cancer therapeutic. Consistent with our findings, Regulus Therapeutics (http://regulusrx.com) is recruiting patients for phase 1 testing of a miR-10b inhibitor to treat patients with GBM. These data show that inhibition of endogenous miR-10b-5p can serve to normalize 5hmC changes in GBM to inhibit neoplastic growth by increasing TET2 expression and we may be seeing these molecular therapeutics in the clinic sooner than anticipated.

**Fig. 6:**
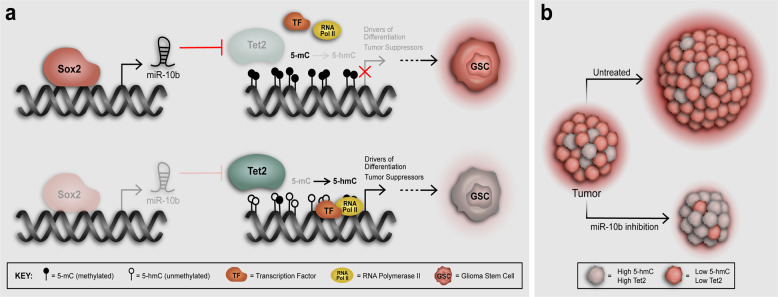
Mechanism by which distinct reprogramming transcription factors (i.e., OCT4 and SOX2) modulate the epigenetic landscape to alter downstream gene targets in GSCs. **a** SOX2 directly transactivates miR-10b-5p resulting in decreased levels of TET2 and decreased conversion of 5mC to 5hmC by TET2. This loss of TET2 associates with and drives tumor-propagating GBM stem cells, presumably by repressing genes that induce cell differentiation, and supports GBM growth. **b** Inhibiting miR-10b-5p expression normalizes TET2 expression, increases 5hmC levels, inhibits GBM-propagating stem cells, induces tumor regression, and prolongs survival. These findings highlight the importance of TET2 and 5hmC loss in SOX2-driven oncogenesis and their potential for therapeutic targeting

The goal of this study was to understand the molecular mechanisms involved in 5hmC deregulation in GBM. We show for the first time that SOX2 represses the TET2 demethylase and decreases 5hmC in GSCs. TET2 repression and 5hmC reduction is sufficient to enhance self-renewal capacity and tumor growth capacity of GSCs. We also show that SOX2 directly activates miR-10b-5p and identify TET2 as its main target in GSCs. Inhibiting miR-10b-5p partially rescues the reduction in 5hmC expression observed in GSCs expressing transgenic SOX2, thus implicating miR-10b-5p as a critical mediator of SOX2-induced onco-methylation, GSC induction and glioma malignancy. Importantly, repression of miR-10-5p enhances TET2 and 5hmC levels in GSCs and blocks tumor-initiation capacity of GSCs and prolongs survival in orthotopic GBM xenograft models. Our results support a mechanism in which SOX2 represses TET2 leading to 5hmC loss in GSCs and miR-10b-5p functions as a key intermediary of this process (Fig. 6). Our findings demonstrate that targeting this novel mechanism of epigenetic dysregulation by inhibiting miR-10b-5p in vivo can lead to pre-clinical GBM therapeutics.

## Materials and methods

### Cell culture

GBM-derived neurosphere lines (GBM1A and GBM1B) were originally derived and characterized by Vescovi et al.^62^ Low-passage primary neurospheres were derived directly from human GBM clinical specimens obtained during clinically indicated surgeries at Johns Hopkins Hospital using established methods.^5^ All GSCs utilized in this study are models of primary GBM (IDH wild-type). The human GBM xenograft line, Mayo39, was originally obtained from the Mayo Clinic (Rochester, MN).^63^ All neurospheres were cultured in serum-free conditions using Stemline(R) Neural Stem Cell Expansion Medium (Sigma-Millipore) supplemented with 20 ng/mL epidermal growth factor and 10 ng/mL fibroblast growth factor. The human embryonic kidney 293FT (HEK293FT) cell line was obtained from the ATCC and was maintained in Dulbecco’s modified Eagle/F12 medium (1:1, vol/vol) supplemented with 10% FBS (Fetal Bovine Serum, Thermo Fisher Scientific Inc, Waltham, MA). All cells were grown at 37 °C in a humidified incubator with 5% CO_2_. All cell lines used in the study were tested for mycoplasma and were STR profiled.

### qRT-PCR and miRNA expression

Total RNA was extracted from cells using RNeasy Mini Kit (Qiagen). cDNA was made by reverse-transcribing 1 μg of total RNA using MuLV Reverse Transcriptase and Oligo (dT) primers (Applied Biosystems). qRT-PCR was performed with a Bio-Rad CFX Detection System (Bio-Rad) and expression of target genes was measured using Power SYBR green PCR kit (Applied Biosystems). Samples were amplified in triplicate and relative gene expression was analyzed using Bio-Rad CFX manager software and normalized to 18S RNA. Primer sequences were used to measure the expression of reprogramming transcription factors, stem cell and neural lineage markers were previously reported by us.^21^ Primer sequences used in this study were obtained from PrimerBank (https://pga.mgh.harvard.edu/primerbank/) and are listed in Supplementary Tables 1 and 2.

### Chromatin immunoprecipitation

Chromatin immunoprecipitation assays were performed using the MAGnify Chromatin Immunoprecipitation System (Life Technologies, Grand Island, NY, USA). Immunoprecipitation was performed with, anti-Sox2 (Cell Signaling Technologies), or anti-IgG (Life Technologies, Grand Island, NY, USA). Specific regions were quantified by qRT-PCR using primers described in the Supplementary Table 3.

### Luciferase reporter assay

The putative miR-10b-5p promoter regions containing the Sox2 binding sites validated in our ChIP experiments were amplified from genomic DNA isolated from GBM1A neurospheres. PCR products were cloned into the XhoI and BglII sites of the pGL4.2 vector (Promega) and verified using Sanger sequencing. 293T or GBM neurospheres were transfected with the indicated reporter constructs using Lipofectamine 3000 (Thermo Fisher Scientific) and luciferase activity was measured using a Luciferase assay kit (Promega) 48 h after transfection. Primers used for cloning can be found in Supplementary Table 4.

Site-directed mutagenesis was performed using the pGL4-TET2′UTR plasmid. The PCR amplifications were carried out with Platinum™ PCR SuperMix High Fidelity PCR system (Thermo Fisher Scientific). The 50 μL PCR reaction was carried out with 100 ng templates, 1 μM primer pair, 200 μM dNTPs, and 2 U of DNA polymerase. The extension reaction was initiated by pre-heating the reaction mixture to 94 °C for 2 min; 16 cycles of 94 °C for 30 s, 55 °C for 30 s, and 68 °C for 16 min. The PCR-amplification products were purified using the QIAquick™ PCR purification kit (Qiagen, Germany) and treated with the restriction enzyme DpnI (New England Biolabs) for 2 h. The PCR product treated with DpnI was transformed into DH5-α chemo-competent cells (New England Biolabs) and inoculated on Luria–Bertani plate containing 100 μg/mL ampicillin. A total of ten colonies were selected and their plasmids were isolated by mini-prep. The positive mutants were confirmed by sanger sequencing.

### Lentivirus generation and cell transduction

For the production of lentiviral particles, we used the second-generation lentiviral system according to Addgene instructions, using psPAX2 packaging plasmid and pMD2.G envelope plasmid (Addgene, Cambridge, MA). Co-transfection of the lentiviral packaging/envelope plasmids and transfer vector into the HEK239FT (2 × 10^7^ cells/transfection) was performed using Lipofectamine 3000 (Thermo Fisher Scientific) scaled according to the manufacturer’s recommendations. After overnight incubation, sodium butyrate (Cayman Chemical) was added at a final concentration of 10 mM to increase viral titer. The lentiviral particles in the supernatant were collected at 48–72 h and used to transduce cells. GBM neurospheres (1.5 × 10^5^ cells) were seeded in a 6-well cell culture plate and infected overnight with a lentiviral medium containing viral particles and polybrene (1 µg/mL), supplemented with the appropriate medium. On the following morning, cells were pelleted by centrifugation and resuspended in a fresh neurosphere medium. A list of lentiviral constructs used can be found in Supplementary Table 5.

### Immunoblotting

Western blot was performed using a quantitative Western-Blot System (LI-COR Bioscience, Lincoln, NE, USA) following the manufacturer’s instructions. Cells were lysed in RIPA buffer (Sigma-Millipore) for 30 min on ice. Samples containing identical amounts of protein (25–40 µg) were resolved by NOVEX 4–12% Tris-glycine gradient gel (Thermo Fisher Scientific), transferred to Amersham Protran nitrocellulose membrane (GE HealthCare), and blocked in Li-COR blocking buffer. Membranes were probed with antibodies listed in Supplementary Table 6. Secondary antibodies were labeled with IRDye infrared dyes (LI-COR Biosciences) and protein levels were quantified using the Odyssey Infrared Imager (LI-COR Biosciences). Densitometry analysis was performed using the Image Studio™ acquisition software from LI-COR imaging systems. Protein expression was normalized to the loading control (i.e., Actin).

### Dot blot analysis of DNA

Dot blot analysis was performed as described by Brown et al.^64^ Briefly, genomic DNA was obtained from GBM neurospheres using the QIAamp DNA Mini Kit (Qiagen). Then, 500 ng of genomic DNA was mixed with 6× SSC buffer and DNA was denatured by incubating at 100 °C for 10 min. The 6× SSC buffer containing the genomic DNA was then placed on ice for 2 min prior to spotting onto nitrocellulose membrane using the Bio-Rad Bio-Dot SF manifold. The membranes were then soaked in Denaturing solution (1.5 M NaCl/0.5 M NaOH) for 10 min followed by incubation in neutralizing solution (1 M NaCl/0.5 M Tris⋅Cl, pH 7.0) for 5 min. After blotting dry using Whatman 3MM filter paper, membranes were blocked in Li-COR blocking buffer and then probed with antibodies against 5mC (Active motif), 5hmC (Active motif), or double-stranded DNA (dsDNA, Abcam). Secondary antibodies were labeled with IRDye infrared dyes (LI-COR Biosciences) and DNA levels were quantified using the Odyssey Infrared Imager (LI-COR Biosciences). Densitometry analysis was performed using the Image Studio™ acquisition software from LI-COR imaging systems. 5mC or 5hmC expression was normalized to dsDNA.

### Intra-cranial nano-miR delivery and tumor formation in vivo

A transcranial cannula was placed so that the tip is in the right caudate/putamen of female athymic nude NCR Nu/Nu mice (8-week old). One week after cannula placement, animals received 1.0 × 10^4^ tumor-propagating cells via the cannula and were assigned to different treatment groups in a non-blinded, randomized manner. Using the same cannula, the control cohort received nano-miRs loaded with control miRNA labeled with Dy547 (IP-004500-01-05) and the experimental group received nano-miRs loaded with the miR-10b-5p inhibitor (IH-300550-08-0005) obtained from Horizon Discovery Ltd.

Stainless steel guide and dummy cannulas were custom ordered from PlasticsOne (Roanoke, VA). The guide cannula (26 gauge) was designed to have a Decon^®^ mesh under the pedestal and cut 3 mm from the mesh. The guide cannula is capped with a screw-on dummy cannula 6.5 mm long so that a 0.5-mm projection extends past the guide to prevent blockage. Prior to surgical placement of cannulas, mice were anesthetized using a Ketamine (100 mg/kg)/Xylazine (10 mg/kg) cocktail and mounted on a stereotactic frame. A rostro-caudal incision was made with a scalpel, the skin spread apart, the surface of the skull was exposed, and cannulas were placed at coordinates: AP (antero-posterior) 0.0 (0 mm from bregma), L (lateral) 1.8 (1.8 mm right from mid-sagittal line).

Lyophilized and resuspended nano-miRs were slowly infused (5 μL) into the brains (0.5 μL/min with a 2 min wait at the end) twice a week as described for each experiment. At the end of the experiment, animals were anesthetized and then sacrificed by perfusion using 4% paraformaldehyde (PFA) according to methods approved by the Animal Use and Care Committee at Johns Hopkins University. All the sectioning and histological analysis were performed in-house. Whole brains were collected and soaked in 4% PFA for 2 days then washed 1× with PBS and soaked in 30% sucrose overnight at 4 °C then flash frozen using dry ice. Brains were embedded in Tissue-Tek^®^ O.C.T. Compound (VWR, Radnor, PA) and 20-μm sections were cut using the CryoStat system from Microm (Walldorf, Germany).

Tumor growth inhibition was determined by computer-assisted morphometric quantification of tumor area in H&E-stained histologic sections using ImageJ software and volumes calculated using volume = (square root of maximum cross-sectional area).^3^ Data for all in vivo experiments are shown as the mean tumor volume distribution of all animals used in the study. All animal procedures were approved by the Johns Hopkins Institutional Animal Care and Use Committee (Protocol# MO14M307), and were in accordance with the NIH Guide for the Care and Use of Laboratory Animals.

### Patient databases

Clinical and transcriptomic data from control and glioma patient samples for TET2 was retrieved from GlioVis database (http://gliovis.bioinfo.cnio.es/) and miR-10b-5p from BetaStasis (https://www.betastasis.com). TET2 expression and survival correlations were determined in primary, IDH wild-type GBM patients. For survival analyses, expression cut-offs were set at 2.61 for CCGA; 7.76 for Gravendeel; 9.2 for TCGA-RNA-Seq; and 5.48 for REMBRANDT. For statistical analysis, pairwise comparisons between group levels with corrections for multiple testing (*p* values with Bonferroni correction) were used. Statistical significance was calculated using unpaired, non-parametric, Student’s *t*-test with Mann–Whitney post hoc test. Clinical and pathological information of specimens included in the analysis is summarized in Supplementary Table 7.

### Statistical analysis

All experiments were performed in triplicates and repeated at least twice in each cell model (*N* ≥ 6). PRISM GraphPad 9 was used to perform all the statistical analyses presented. Two group comparisons were analyzed for variation and significance using a two-tailed, type 1 *t*-test and *p* values lower than 0.05 were considered significant and symbolized by an asterisk in the graphs. One-way or two-way ANOVA and Tukey or Bonferroni post hoc tests were used to analyze the relationships when comparing multiple variables, with *p* values lower than 0.05 considered to be statistically significant. All data shown are representative of mean ± SD of triplicate results unless otherwise specified.

## Supplementary information

Supplementary materials

## Data Availability

The data used for the current study are available from the corresponding author upon request.

## References

[1] Easwaran H, Tsai HC, Baylin SB (2014). Cancer epigenetics: tumor heterogeneity, plasticity of stem-like states, and drug resistance. Mol. Cell.

[2] Baylin SB, Ohm JE (2006). Epigenetic gene silencing in cancer—a mechanism for early oncogenic pathway addiction?. Nat. Rev. Cancer.

[3] Kazanets A (2016). Epigenetic silencing of tumor suppressor genes: paradigms, puzzles, and potential. Biochim Biophys. Acta.

[4] Rajendran G (2011). Epigenetic regulation of DNA methyltransferases: DNMT1 and DNMT3B in gliomas. J. Neurooncol..

[5] Mercher T (2012). TET2, a tumor suppressor in hematological disorders. Biochim. Biophys. Acta.

[6] Chen ZX, Riggs AD (2011). DNA methylation and demethylation in mammals. J. Biol. Chem..

[7] Tahiliani M (2009). Conversion of 5-methylcytosine to 5-hydroxymethylcytosine in mammalian DNA by MLL partner TET1. Science.

[8] Kudo Y (2012). Loss of 5-hydroxymethylcytosine is accompanied with malignant cellular transformation. Cancer Sci..

[9] Thienpont B, Galle E, Lambrechts D (2016). TET enzymes as oxygen-dependent tumor suppressors: exciting new avenues for cancer management. Epigenomics.

[10] Ostrom QT (2016). American Brain Tumor Association Adolescent and Young Adult Primary Brain and Central Nervous System Tumors Diagnosed in the United States in 2008-2012. Neuro Oncol..

[11] Bao S (2006). Glioma stem cells promote radioresistance by preferential activation of the DNA damage response. Nature.

[12] Lathia JD (2015). Cancer stem cells in glioblastoma. Genes Dev..

[13] Orr BA (2012). Decreased 5-hydroxymethylcytosine is associated with neural progenitor phenotype in normal brain and shorter survival in malignant glioma. PLoS One.

[14] Zhou D (2018). Distinctive epigenomes characterize glioma stem cells and their response to differentiation cues. Genome Biol..

[15] Maleszewska M, Kaminska B (2013). Is glioblastoma an epigenetic malignancy?. Cancers.

[16] Zhang F (2016). 5-hydroxymethylcytosine loss is associated with poor prognosis for patients with WHO grade II diffuse astrocytomas. Sci. Rep..

[17] Kraus TF (2015). Genetic characterization of ten-eleven-translocation methylcytosine dioxygenase alterations in human glioma. J. Cancer.

[18] Kraus TF (2015). Loss of 5-hydroxymethylcytosine and intratumoral heterogeneity as an epigenomic hallmark of glioblastoma. Tumour Biol..

[19] Figueroa ME (2010). Leukemic IDH1 and IDH2 mutations result in a hypermethylation phenotype, disrupt TET2 function, and impair hematopoietic differentiation. Cancer Cell.

[20] Turkalp Z, Karamchandani J, Das S (2014). IDH mutation in glioma: new insights and promises for the future. JAMA Neurol..

[21] Lopez-Bertoni H (2015). DNMT-dependent suppression of microRNA regulates the induction of GBM tumor-propagating phenotype by Oct4 and Sox2. Oncogene.

[22] Malta TM (2018). Machine learning identifies stemness features associated with oncogenic dedifferentiation. Cell.

[23] Lopez-Bertoni H (2018). Bioreducible polymeric nanoparticles containing multiplexed cancer stem cell regulating miRNAs inhibit glioblastoma growth and prolong survival. Nano Lett..

[24] Dong Z (2019). Targeting glioblastoma stem cells through disruption of the circadian clock. Cancer Disco..

[25] Darmanis S (2017). Single-cell RNA-seq analysis of infiltrating neoplastic cells at the migrating front of human glioblastoma. Cell Rep..

[26] Munoz P, Iliou MS, Esteller M (2012). Epigenetic alterations involved in cancer stem cell reprogramming. Mol. Oncol..

[27] Hysolli E (2016). Regulation of the DNA methylation landscape in human somatic cell reprogramming by the miR-29 family. Stem Cell Rep..

[28] Singh SK (2004). Identification of human brain tumour initiating cells. Nature.

[29] Sato F, Tsuchiya S, Meltzer SJ, Shimizu K (2011). MicroRNAs and epigenetics. FEBS J..

[30] Cheng J (2013). An extensive network of TET2-targeting MicroRNAs regulates malignant hematopoiesis. Cell Rep..

[31] Lopez-Bertoni H (2016). Epigenetic modulation of a miR-296-5p:HMGA1 axis regulates Sox2 expression and glioblastoma stem cells. Oncogene.

[32] Lopez-Bertoni H (2020). A Sox2/miR-486-5p axis regulates survival of GBM cells by inhibiting tumor suppressor networks. Cancer Res..

[33] Kraus TF (2012). Low values of 5-hydroxymethylcytosine (5hmC), the “sixth base,” are associated with anaplasia in human brain tumors. Int J. Cancer.

[34] Garcia MG (2018). Epigenetic dysregulation of TET2 in human glioblastoma. Oncotarget.

[35] Slack FJ, Chinnaiyan AM (2019). The role of non-coding RNAs in oncology. Cell.

[36] Zhu Q (2016). miR-10b exerts oncogenic activity in human hepatocellular carcinoma cells by targeting expression of CUB and sushi multiple domains 1 (CSMD1). BMC Cancer.

[37] Bahena-Ocampo I (2016). miR-10b expression in breast cancer stem cells supports self-renewal through negative PTEN regulation and sustained AKT activation. EMBO Rep..

[38] Ye P (2015). Enhancing HOTAIR/MiR-10b drives normal liver stem cells toward a tendency to malignant transformation through inducing epithelial- to-mesenchymal transition. Rejuvenation Res..

[39] Guessous F (2013). Oncogenic effects of miR-10b in glioblastoma stem cells. J. Neurooncol..

[40] He C (2015). Demethylation of miR-10b plays a suppressive role in ccRCC cells. Int J. Clin. Exp. Pathol..

[41] Li Z (2015). DNA methylation downregulated mir-10b acts as a tumor suppressor in gastric cancer. Gastric Cancer.

[42] Yu M (2018). miR-10b downregulated by DNA methylation acts as a tumor suppressor in HPV-positive cervical cancer via targeting Tiam1. Cell Physiol. Biochem..

[43] Wimmer M (2020). A cancer stem cell-like phenotype is associated with miR-10b expression in aggressive squamous cell carcinomas. Cell Commun. Signal.

[44] Teplyuk NM (2016). Therapeutic potential of targeting microRNA-10b in established intracranial glioblastoma: first steps toward the clinic. EMBO Mol. Med..

[45] Gabriely G (2011). Human glioma growth is controlled by microRNA-10b. Cancer Res..

[46] Sun B (2019). Stepwise detection and evaluation reveal miR-10b and miR-222 as a remarkable prognostic pair for glioblastoma. Oncogene.

[47] Jeschke J, Collignon E, Fuks F (2016). Portraits of TET-mediated DNA hydroxymethylation in cancer. Curr. Opin. Genet Dev..

[48] Thomson JP, Meehan RR (2017). The application of genome-wide 5-hydroxymethylcytosine studies in cancer research. Epigenomics.

[49] Chen B (2017). Repression of the expression of TET2 by ZEB1 contributes to invasion and growth in glioma cells. Mol. Med. Rep..

[50] Song SJ (2013). The oncogenic microRNA miR-22 targets the TET2 tumor suppressor to promote hematopoietic stem cell self-renewal and transformation. Cell Stem Cell.

[51] Fernandez AF (2018). Loss of 5hmC identifies a new type of aberrant DNA hypermethylation in glioma. Hum. Mol. Genet.

[52] Pangeni RP (2018). Genome-wide methylomic and transcriptomic analyses identify subtype-specific epigenetic signatures commonly dysregulated in glioma stem cells and glioblastoma. Epigenetics.

[53] Rodger EJ, Chatterjee A, Morison IM (2014). 5-hydroxymethylcytosine: a potential therapeutic target in cancer. Epigenomics.

[54] Cimmino L (2017). Restoration of TET2 function blocks aberrant self-renewal and leukemia progression. Cell.

[55] Tucker DW (2018). Epigenetic reprogramming strategies to reverse global loss of 5-hydroxymethylcytosine, a prognostic factor for poor survival in high-grade serous ovarian cancer. Clin. Cancer Res..

[56] Cui Q (2016). Downregulation of TLX induces TET3 expression and inhibits glioblastoma stem cell self-renewal and tumorigenesis. Nat. Commun..

[57] Gonzalez-Aseguinolaza G, Givosiran - (2020). Running RNA interference to fight porphyria attacks. N. Engl. J. Med..

[58] Urits I (2021). A review of patisiran (ONPATTRO(R)) for the treatment of polyneuropathy in people with hereditary transthyretin amyloidosis. Neurol. Ther..

[59] Alzhrani R (2020). Improving the therapeutic efficiency of noncoding RNAs in cancers using targeted drug delivery systems. Drug Disco. Today.

[60] Lopez-Bertoni H, Laterra J (2021). Opinion: miRNAs – the new wave of molecular cancer therapeutics. Transl. Oncol..

[61] Ren S, Xu Y (2019). AC016405.3, a novel long noncoding RNA, acts as a tumor suppressor through modulation of TET2 by microRNA-19a-5p sponging in glioblastoma. Cancer Sci..

[62] Galli R (2004). Isolation and characterization of tumorigenic, stem-like neural precursors from human glioblastoma. Cancer Res..

[63] Pandita A (2004). Contrasting in vivo and in vitro fates of glioblastoma cell subpopulations with amplified EGFR. Genes Chromosomes Cancer.

[64] Brown, T. Dot and slot blotting of DNA. *Curr. Protoc. Mol. Biol.* **Chapter 2**, Unit2 9B (2001). 10.1002/0471142727.mb0209bs21 18265189

