# Supplementary material for: Sox2 induces glioblastoma cell stemness and tumor propagation by repressing TET2 and deregulating 5hmC and 5mC DNA modifications
Source: Signal Transduct Target Ther. 2022 Feb 9;7:37. doi: 10.1038/s41392-021-00857-0 (PMC8826438; doi:10.1038/s41392-021-00857-0)
Supplement: Supplementary file 1 — Supplementary materials [file 41392_2021_857_MOESM1_ESM.docx]

Supplementary Materials for

**Sox2 induces glioblastoma cell stemness and tumor propagation by repressing TET2 and deregulating 5hmC and 5mC DNA modifications**

*Hernando Lopez-Bertoni^2^, Amanda Johnson, Yuan Rui, Bachchu Lal_,_ Sophie Sall, Maureen Malloy, Johnathan Coulter, Maria Lugo-Fagundo, Sweta Shudir, Harmon Khela, Christopher Caputo, Jordan J. Green and John Laterra^*^*

Correspondence to: Laterra@kennedykrieger.org or Lopezbertoni@kennedykrieger.org

**This PDF file includes:**

Figures. S1 to S13

Tables S1 to S7

Figure. S1.


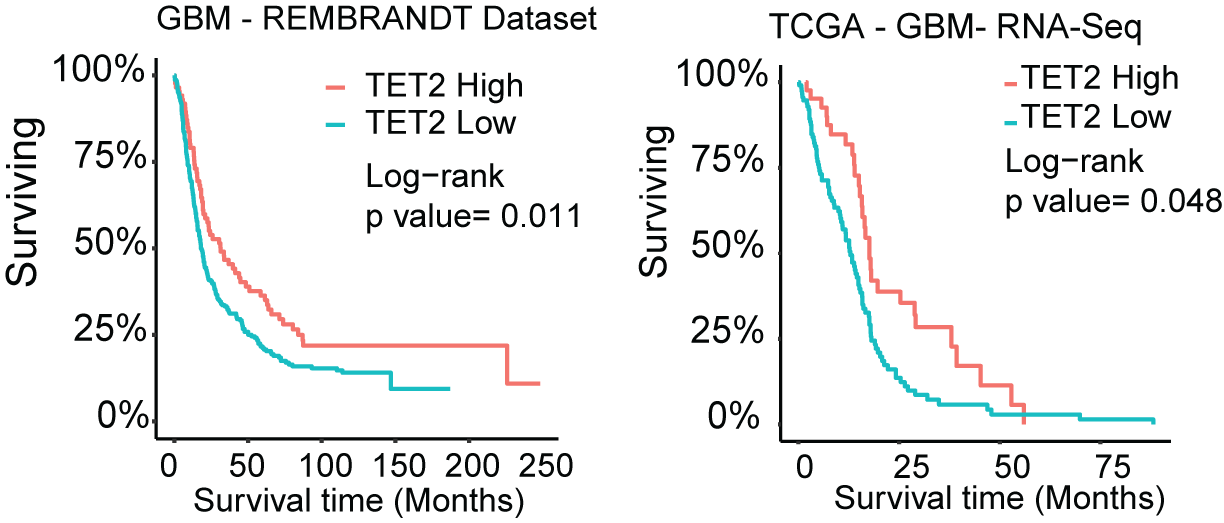


Figure S1: Low TET2 expression associates with poor prognosis in GBM. Kaplan-Meier survival curves comparing GBM patients across multiple datasets. Survival data was retrieved from the GlioVis portal (http://gliovis.bioinfo.cnio.es).

**Figure. S2.**


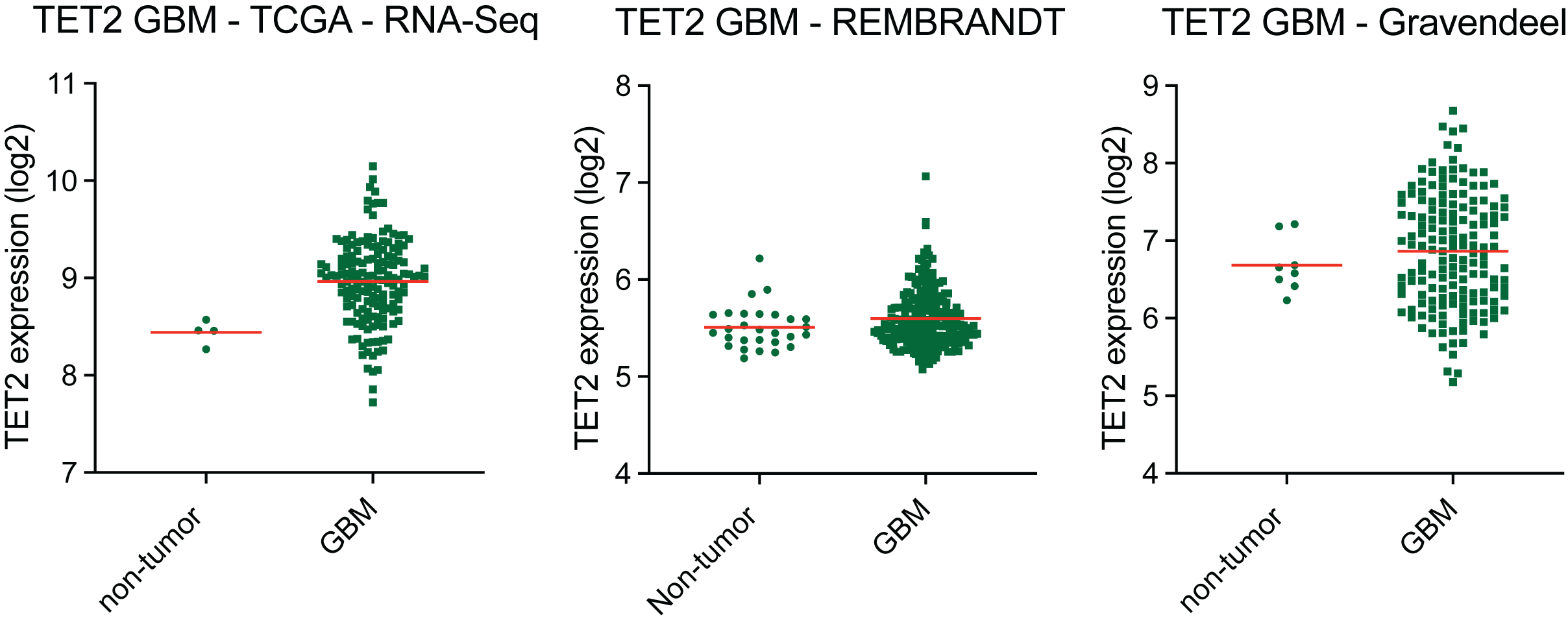
Figure S2: TET2 expression in clinical GBM datasets. (A) TET2 expression data was retrieved from multiple clinical databases using the GlioVis portal (http://gliovis.bioinfo.cnio.es). TET2 expression compared to normal brain varies between datasets. (B) RNA-Seq data comparing TET2 expression in neural stem cells (NSCs) and glioma stem cells (GSCs).

Figure. S3.


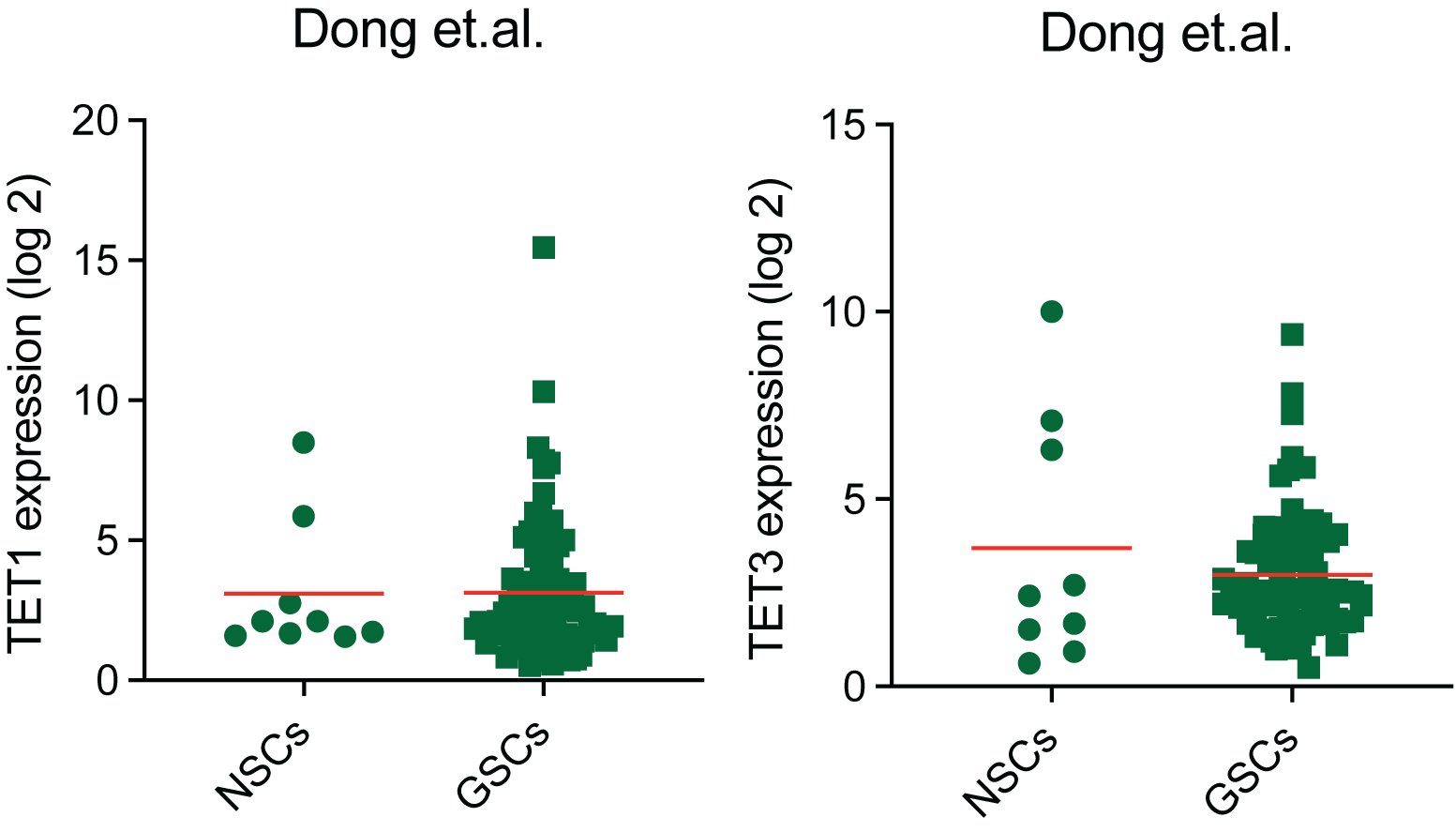


**Figure S3: TET2 expression in GSC datasets**. RNA-Seq data comparing TET2 expression in neural stem cells (NSCs) and glioma stem cells (GSCs). Data was retrieved from Dong *et. al.* (PMID: 31455674; GSE134973)

Figure. S4.


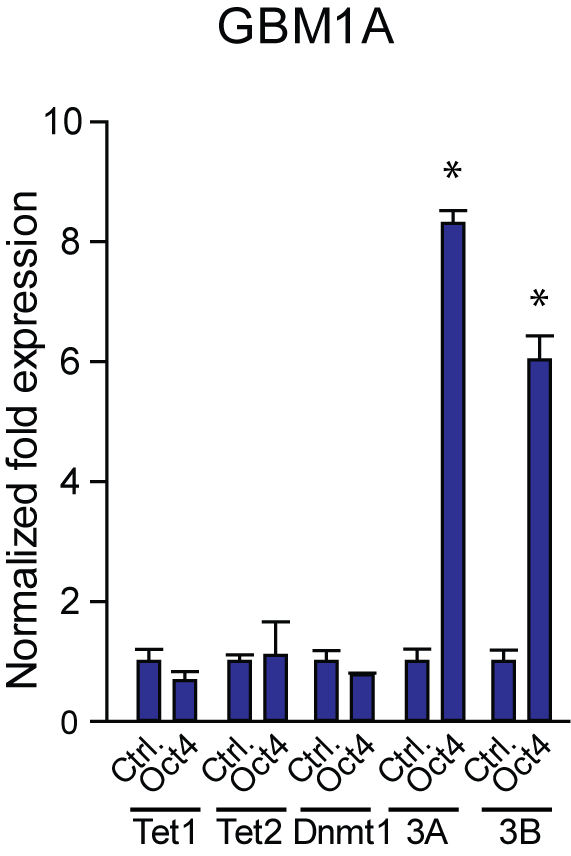


Figure S4: Exogenous Oct4 increases expression of DNMT3A and DNMT3B in GBM cells. qRT-PCR analysis showing selective increase in Dnmt3A and Dnmt3B mRNA in GSCs expressing exogenous Oct4.

Figure. S5.


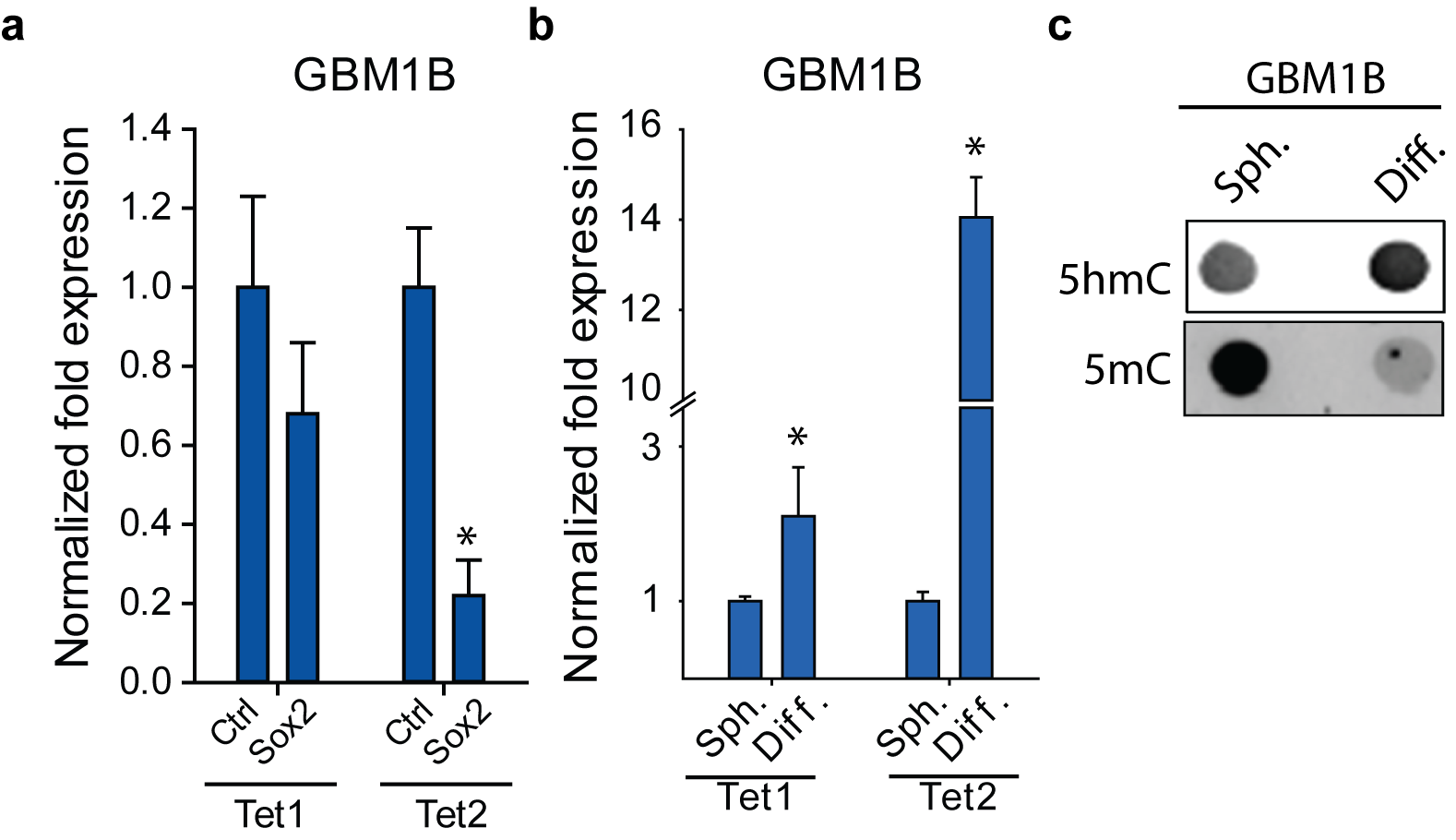


Figure S5: SOX2 decreases Tet2 expression in GSCs. (a) qRT-PCR analysis showing decrease in TET2 mRNA in GSCs expressing exogenous Sox2. (b) qRT-PCR analysis showing increased TET2 mRNA following forced differentiation of GSCs. (c) Dot-blot analysis of genomic DNA isolated from GSCs showing increased 5hmC and decreased 5mC after forced differentiation. Statistical significance was calculated using Student’s t-test in panels a and b and data are presented as means ± S.D. **p*< 0.05

Figure. S6.


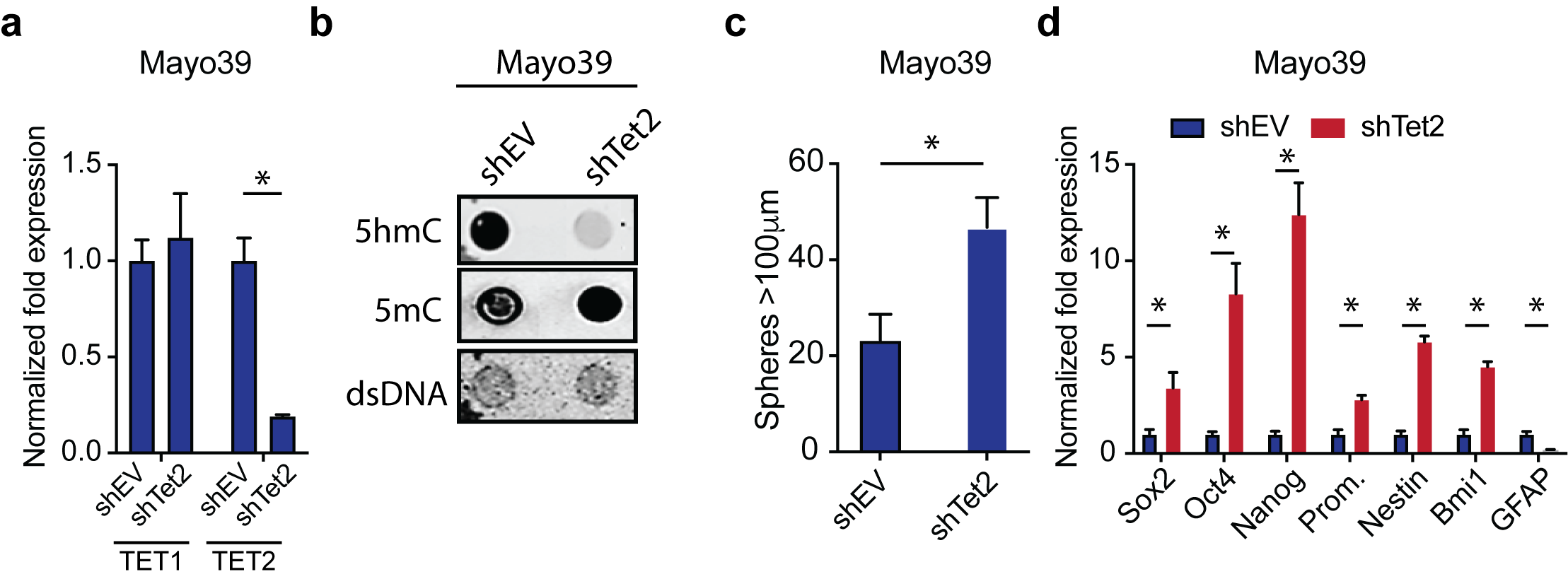


Figure S6: TET2 inhibition enhances the stem cell phenotype of GBM cells. (a) qRT-PCR shows specific knock-down of TET2 expression after transduction of shTET2 construct in patient-derived GSC isolates. (b) Dot blot showing shRNA-mediated inhibition of TET2 reduces 5hmC and increases 5mC in GSC isolates. (c) Equal numbers of GSC isolates transduced with lentiviral constructs expressing two distinct shRNAs targeting TET2 or a control vector (shEV) were cultured in neurosphere medium for 14 days. Quantification of neurospheres (>100µm diameter) by computer-assisted image analysis shows that TET2 knock-down enhances neurosphere formation. (d) qRT-PCR to measure expression of stem cell drivers and markers after TET2 knock-down in GSCs. Statistical significance was calculated using Student’s t-test in panel a, c, and d. Data are presented as means ± S.D **p*< 0.05

Figure. S7.


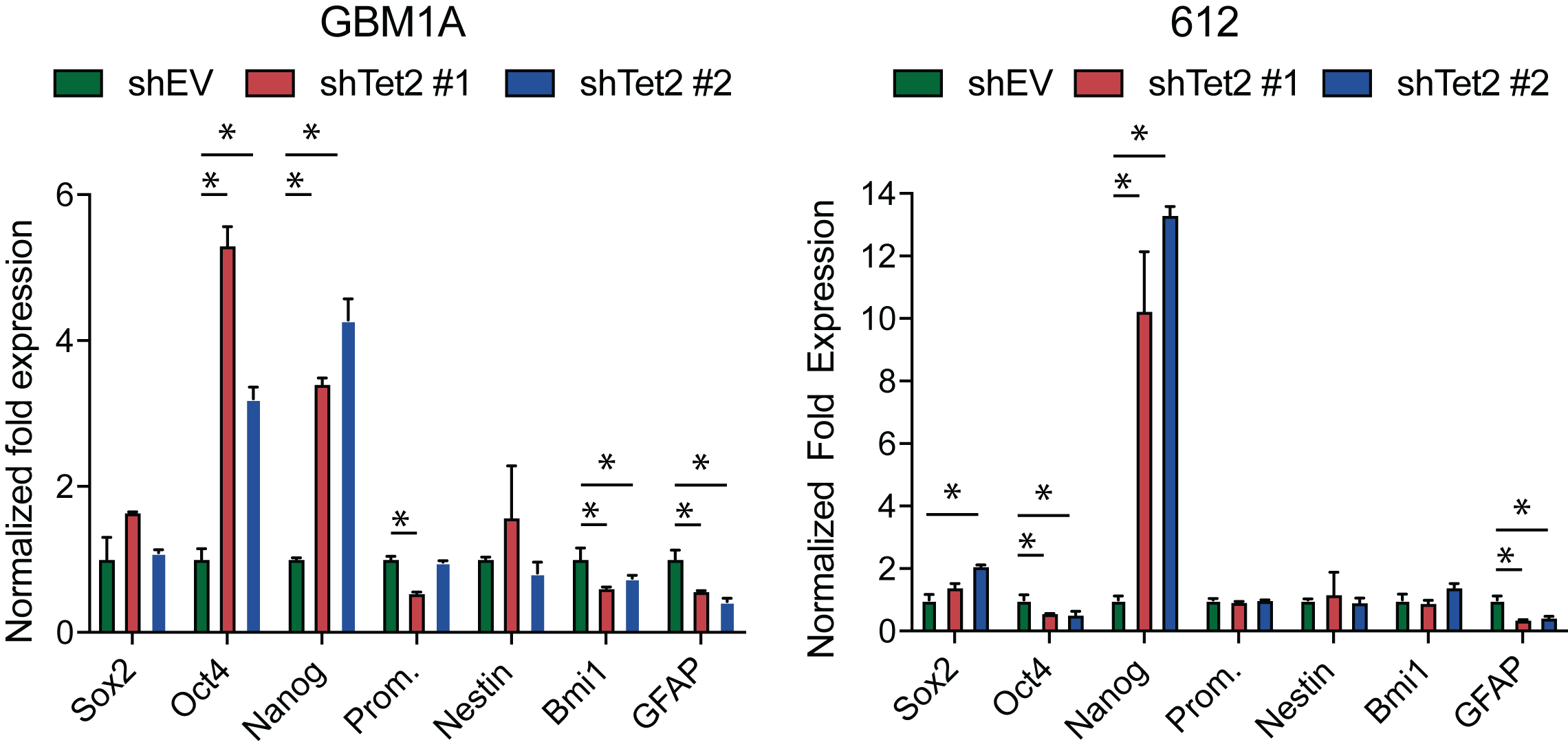


**Figure S7: TET2 inhibition modulates the expression of stem cell markers and drivers in GBM cells.** qRT-PCR to measure expression of stem cell drivers and markers after TET2 knock-down in GSCs. One-way ANOVA with Tuckey’s *post hoc* test was used calculate statistical significance**.** Data are presented as means ± S.D **p*< 0.05


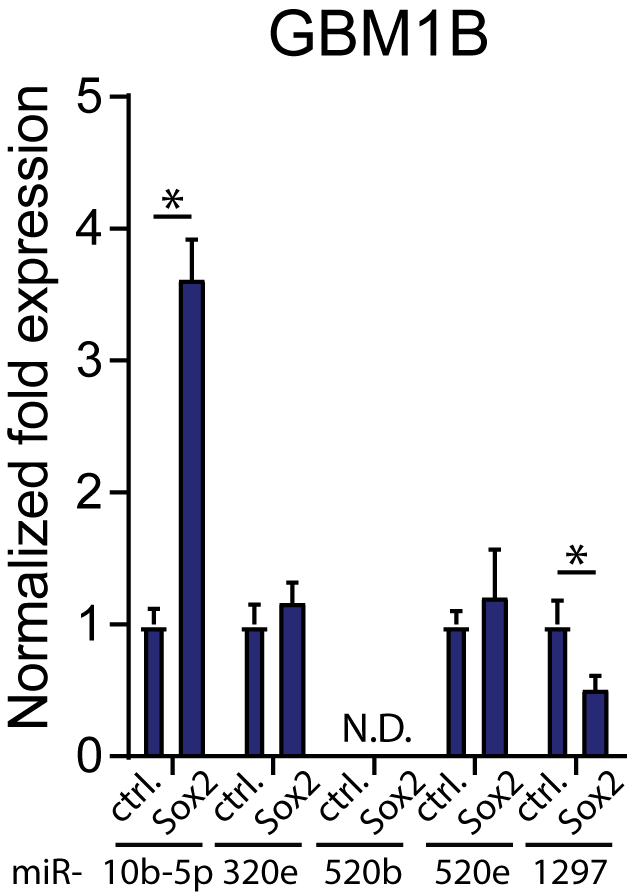
Figure. S8.

**Figure S8: SOX2 induces miR-10b-5p in GSCs**. qRT-PCR to measure expression of pre-cursor miRNAs predicted to inhibit TET2 in GSCs expressing transgenic Sox2.


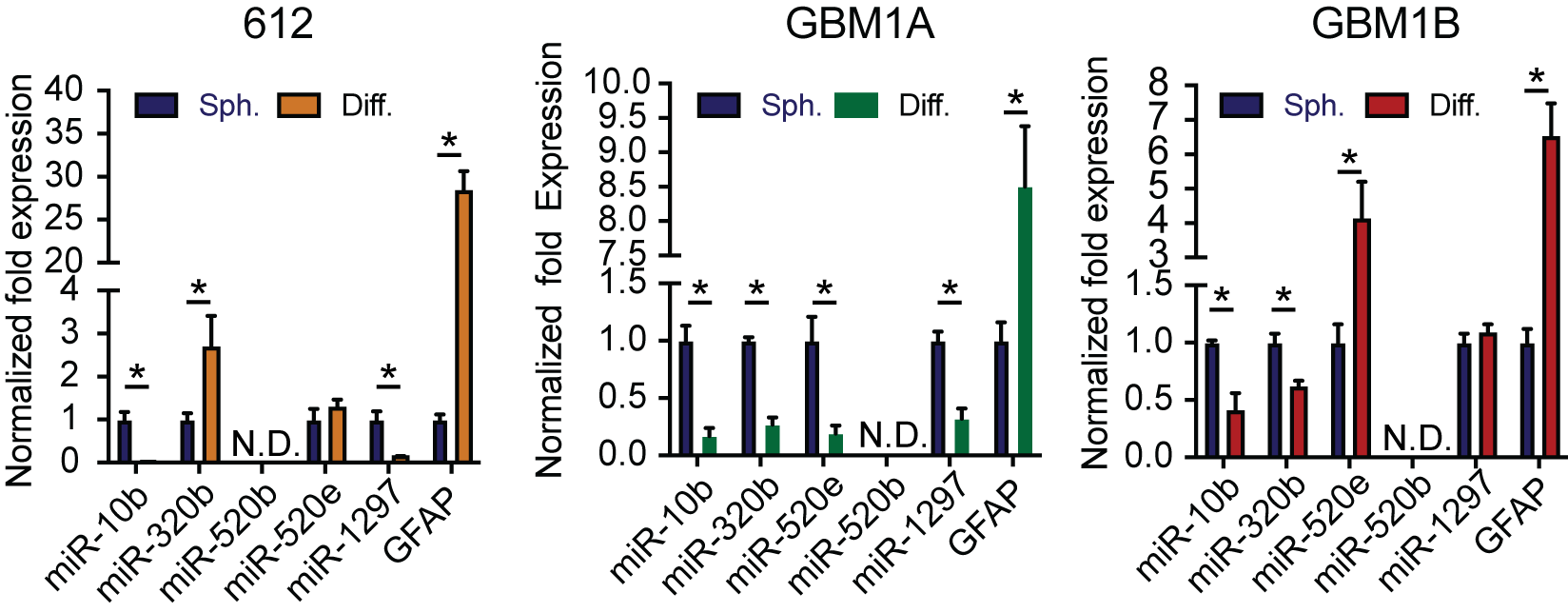
Figure. S9.

Figure S9: miR-10b-5p, a TET2 regulating miRNA, is repressed during GSC differentiation. (A)qRT-PCR to measure expression of miRNAs predicted to target TET2 after forced differentiation of GSCs. Statistical significance was calculated using Student’s t-test. Data are presented as means ± S.D **p*< 0.05

Figure. S10.


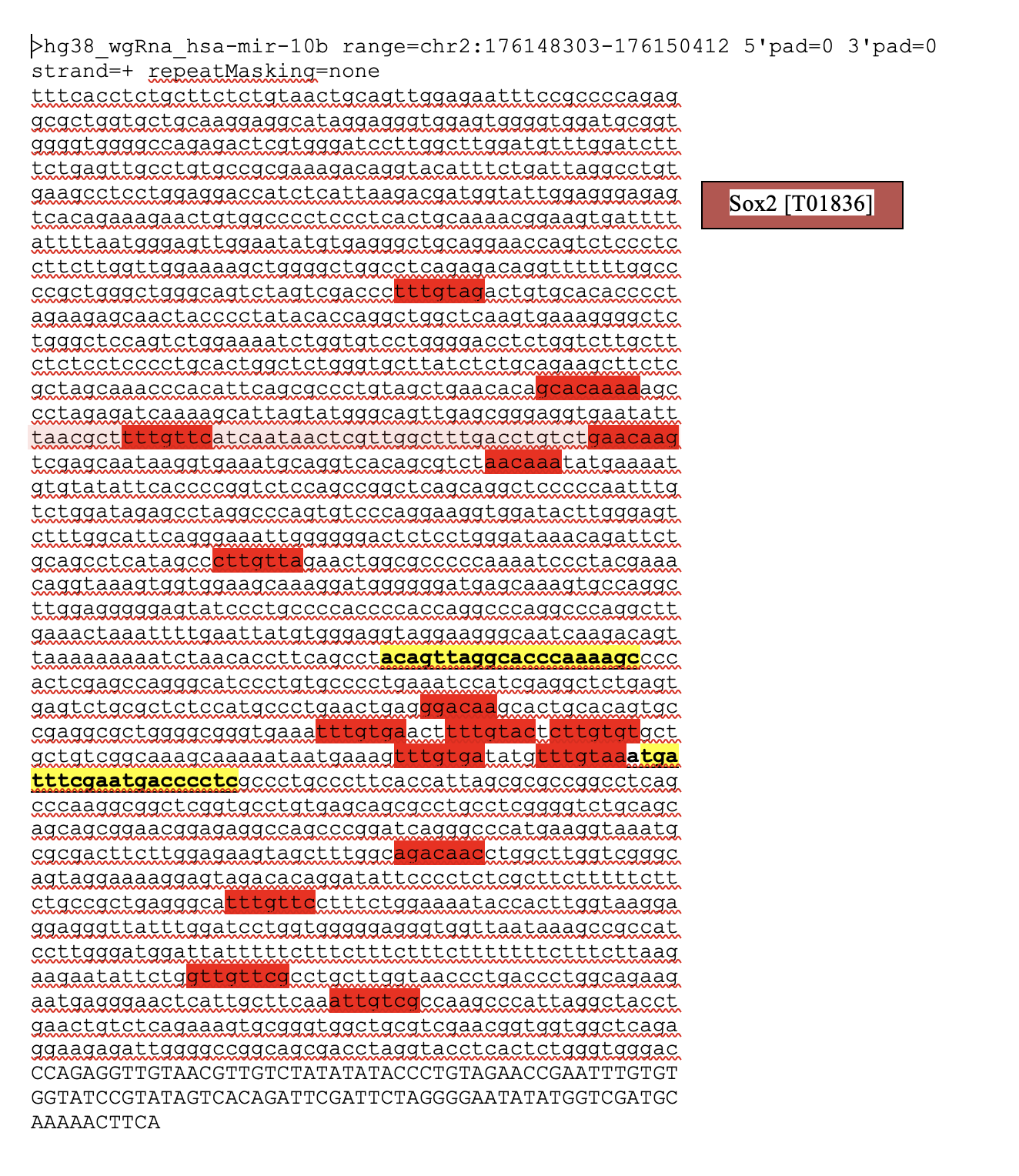


Figure S10: miR-10b-5p 2Kb putative promoter region. Genomic sequence 2kB upstream of the pre-cursor miR-10b (uppercase) was retrieved from the USCS genome browser. Sox2 binding sites (red boxes) were determined using the PROMO web tool (<http://alggen.lsi.upc.es/cgi-bin/promo_v3/promo/promoinit.cgi?dirDB=TF_8.3>). Yellow boxes denote the priming sites used for the ChIP experiments shown in Fig. 3E.

Figure. S11.


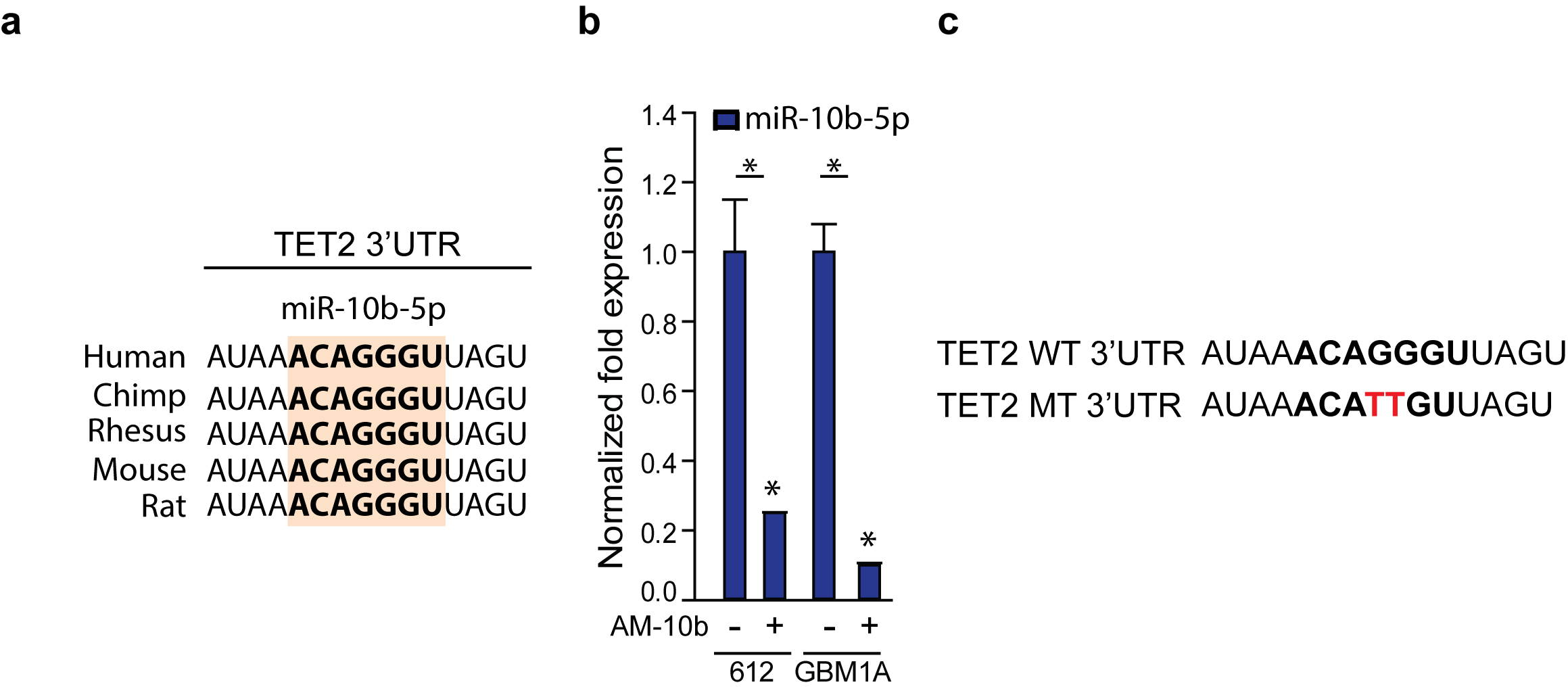


Figure S11: miR-10b-5p regulates TET2 expression in GBM neurospheres. (a) Schematic depicting the miR-10b-5p binding site in the Tet2 3’UTR. (b) qRT-PCR to measure expression of pre-miR-10b-5p 4 days lentiviral transduction. (c) Schematic depicting the mutated miR-10b-5p binding site in the Tet2 3’UTR. Statistical significance was calculated using Student’s t-test. Data are presented as means ± S.D **p*< 0.05

Figure. S12.
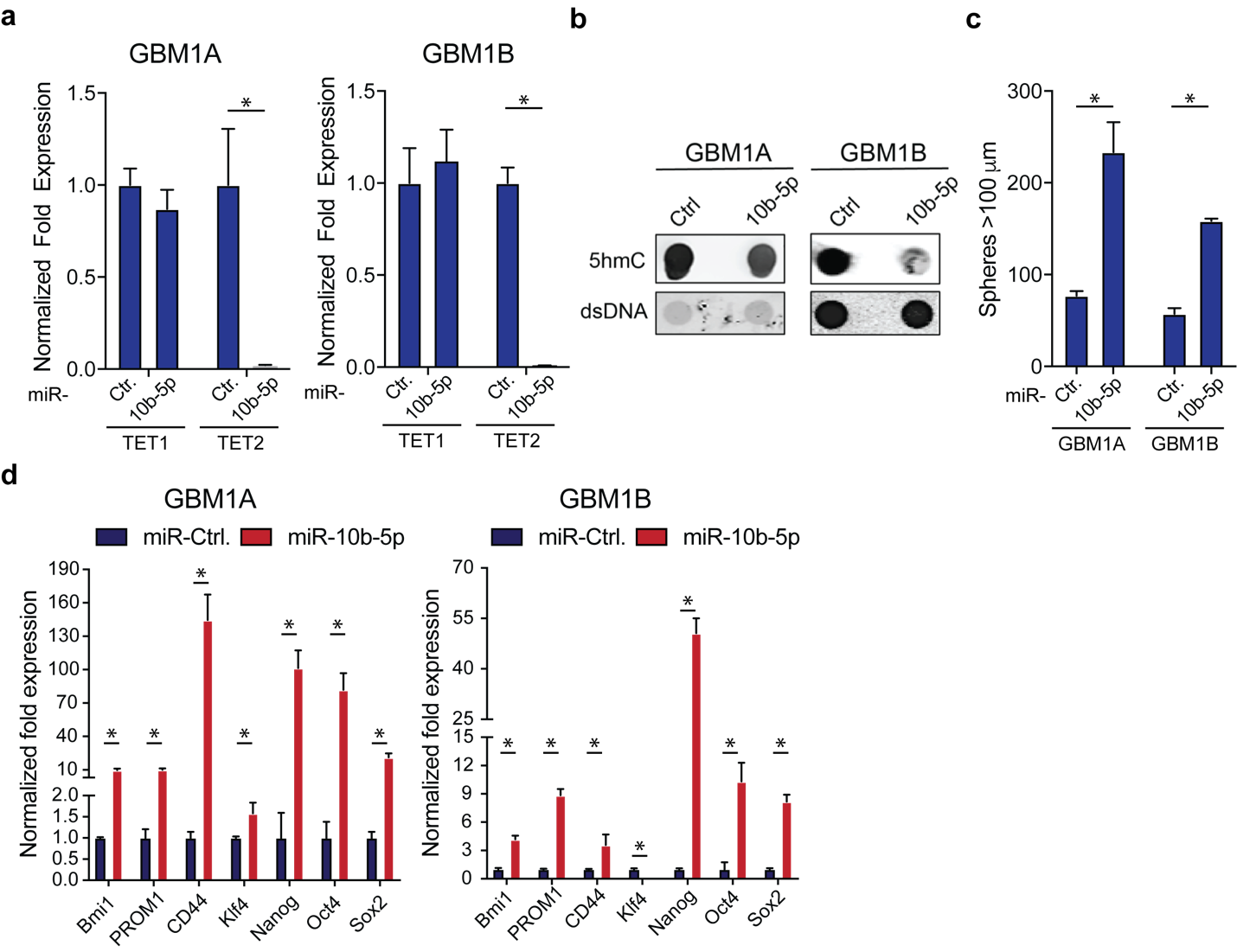


Figure S12: miR-10b-5p inhibits TET2, reduces 5hmC and enhances the stem cell phenotype of GBM cells. (a) qRT-PCR to measure TET1 and TET2 expression after expression of transgenic miR-10b-5p in GSC isolates. (b) Dot blot showing miR-10b-5p-mediated inhibition of TET2 reduces 5hmC and increases 5mC in GSC isolates. (c) Equal numbers of GSC isolates transduced with lentiviral constructs expressing miR-10b-5p or a control vector (miR-Ctrl.) were cultured in neurosphere medium for 14 days. Quantification of neurospheres (>100µm diameter) by computer-assisted image analysis shows that miR-10b-5p expression enhances neurosphere formation. (d) qRT-PCR to measure expression of stem cell drivers and markers after miR-10b-5p expression in GSCs. Statistical significance was calculated using Student’s t-test in panel a, c, and d. Data are presented as means ± S.D **p*< 0.05

Figure. S13.


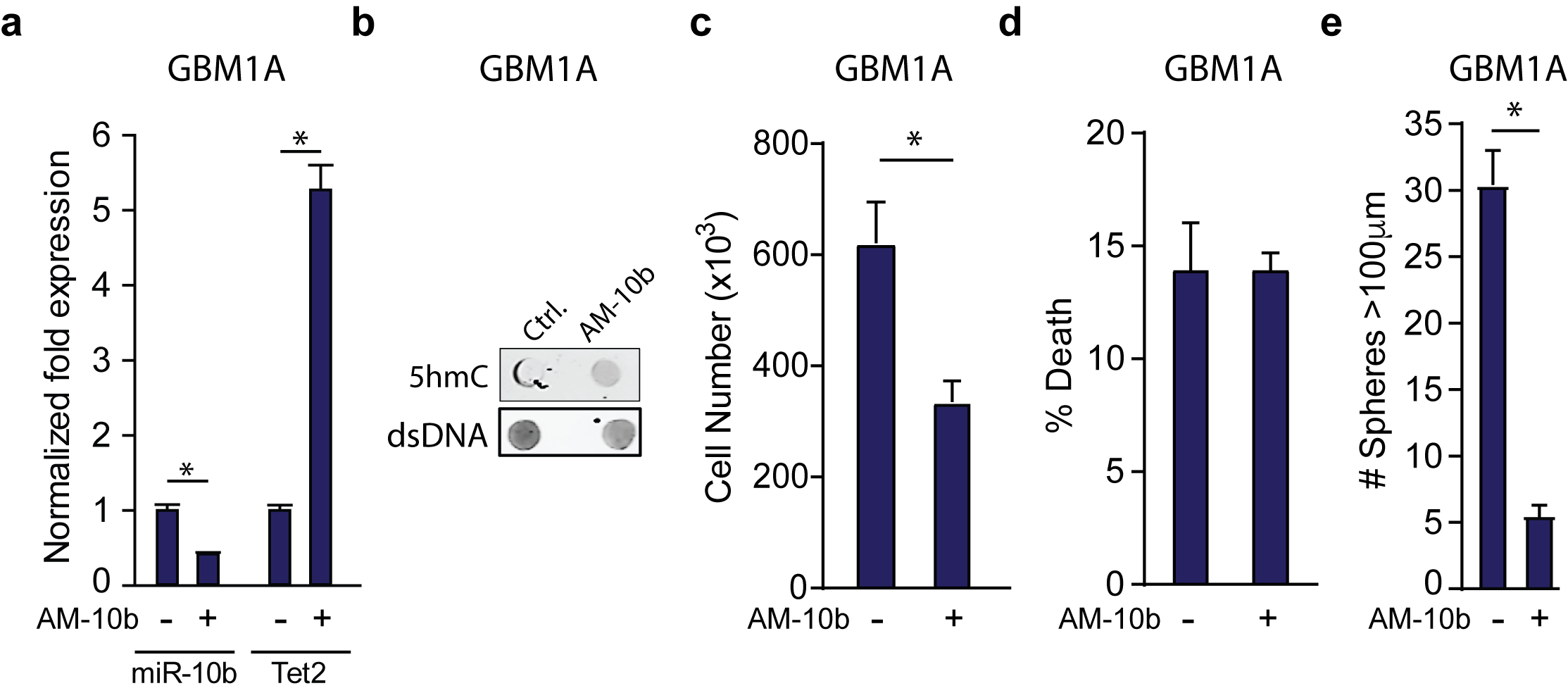


Figure S13: PBAE-mediated delivery of AM-10b-5p inhibits the stem cell phenotype of GBM cells. (a) qRT-PCR analysis to quantify expression of miR-10b-5p and TET2 in GBM neurospheres 3 days after transfection with nano-miRs delivering a non-targeting control miRNA (Ctrl.) or a miR-10b-5p inhibitor (AM-10b-5p). (b) Dot-blot analysis of genomic DNA isolated from neurospheres after miR-10b-5p inhibition . Cell numbers (c) and cell viability (d) was measured 6 days after transfections with nano-miRs delivering a non-targeting control miRNA (Ctrl.) or a miR-10b-5p inhibitor using trypan blue exclusion assay. (e) Equal numbers of GSCs were cultured in neuropshere medium for 14 days and neurosphere-forming capacity was quantified by computer-assisted image analysis. PBAE-mediated miR-10b-5p inhibition decreased sphere-formation capacity of GSCs. Statistical significance was calculated using Student’s t-test in panel a, b, and d. Data are presented as means ± S.D **p*< 0.05

Table S1.

| **Supplemental Table S1: PCR primers used to amplify pre-miRNAs** | | |
| --- | --- | --- |
| **Gene** | **Forward primer (5’ to 3’)** | **Reverse primer (5’ to 3’)** |
| miR-10b-5p | TACCCTGTAGATCCGAATTTGTG | ATTCCCCTAGATACGAATTTGTGA |
| U6 | CTCGCTTCGGCAGCACA | AACGCTTCACGAATTTGCGT |

Table S2.

| **Supplemental Table S2: PCR primers used for qRT-PCR** | | |
| --- | --- | --- |
| **Gene** | **Forward primer (5’ to 3’)** | **Reverse primer (5’ to 3’)** |
| Dnmt1  (NM_001130823) | AAGACAAAGACCAGGATGAGAAG | GGGTGTTGGTTCTTTGGTTTG |
| Dnmt3a  (NM_022552) | TATTGATGAGCGCACAAGAGAGC | GGGTGTTCCAGGGTAACATTGAG |
| Dnmt3b  (NM_006892) | CCATTCGAGTCCTGTCATTG | GCAATGGACTCCTCACACAC |
| TET1 (NM_030625) | GAGCCTGTTCCTCGATGTGG | CAAACCCACCTGAGGCTGTT |
| TET2  (NM_017628) | CACTGCATGTTTGGACTTCTG | TGCTCATCCTCAGGTTTTCC |
| 18S | ACAGGATTGACAGATTGATAGCTC | CAAATCGCTCCACCAACTAAGAA |

**Table S3.**

| **Supplemental Table S3: PCR primers used for ChIP-PCR** | | |
| --- | --- | --- |
| **Gene** | **Forward primer (5’ to 3’)** | **Reverse primer (5’ to 3’)** |
| miR-10b-ChIP2 | gcagtctgcctttgtgttga | aaccagctgcctgtttttgt |
| Detect Sox2 binding to miR-10b-5p promoter – **Sox2 binding site** | | |

**Table S4.**

| **Supplemental Table S4: PCR primers used for luciferase construct** | | |
| --- | --- | --- |
| **Gene** | **Forward primer (5’ to 3’)** | **Reverse primer (5’ to 3’)** |
| Sox2-miR10b luc | CCATGGCTCGAGgcagtctgcctttgtgttga | C CCATGGAGATCTaaccagctgcctgtttttgt |
| TET2-3’UTR_Luc WT | CCATGGCTCGAGgatgcctgcataagatgaataa | CCCATGGAGATCTttaactgacagatttttcttt |
| TET2-3’UTR_Luc MT | gatgcctgcataagatgaataa**acaAAgt**tagttccatgtgaatctgtcagttaa | ttaactgagattcacatggaactaacta**acTTtgt**ttattcatcttatgcag |
| miR-10b-5p promoter region containing the Sox2 binding sites was cloned the XhoI and BglII sites of the pGL4.2 luciferase reporter vector (Promega, Madison, WI - USA). | | |

**Table S5.**

| **Supplemental Table S5: Lentiviral constructs** | | |
| --- | --- | --- |
| **Genecopoeia** | | |
| **Gene** | **Accession #** | **Catalog #** |
| miR-10b-5p mimic | MIMAT0000254 | HmiR0035-MR03 |
| miR-10b-5p Inhibitor | MIMAT0000254 | HmiR-AN0034-AM03 |
| miRNA scrambled control clone for pEZX-MR03 |  | CmiR0001-MR03 |
| **Lentiviral shRNA constructs – Applied Biological Materials Inc. (abm)** | | |
| **Gene** | **Accession #** | **Catalog #** |
| Scrambled siRNA GFP Lentivector |  | LV015-G |
| TET2-425 siRNA/shRNA/RNAi Lentivector (Human)  Set of 4 shRNAs | 465200910495 | NM_017628 |

**Table S6.**

| **Supplemental Table S6: Antibodies** | | |
| --- | --- | --- |
| **Gene** | **Company (Cat.#)** | **Dilution** |
| Tet2 | Bethyl Laboratories, Inc (A304-247A) | 1:250 |
| DNMT3a | Cell Signaling ( 3598 ) | 1:1000 |
| 5mC | Cell Signaling (28692S) | 1:3000 |
| 5hmC | Active motif (39791) | 1:1000 |
| dsDNA | Abcam (ab27156) | 1:1000 |
| Actin | Sigma-Millipore (A1978) | 1:5000 |
| IRDye 800CW Goat anti Rabbit | Li-Cor (926-32211) | 1:10000 |
| IRDye® 680RD Goat anti-Mouse | Li-Cor (926-68070) | 1:10000 |

| **Supplemental Table S7: Sample Sizes & Molecular Features of clinical specimens analyzed** | | | | | |
| --- | --- | --- | --- | --- | --- |
| **Data Set** | | **CCGA** | **Gravendeel** | **TCGA RNA-Seq** | **REMBRANDT** |
| **Histology** | | | | | |
|  | GBM | 388 | 159 | 528 | 219 |
|  | Non-Tumor | 5 | 8 | 10 | 28 |
| **Recurrence** | | | | | |
|  | Primary | 225 | NA | 497 | NA |
|  | Secondary | 30 | NA | 7 | NA |
|  | Recurrent | 134 | NA | 16 | NA |
|  | NA | 102 | NA | 8 | NA |
| **Subtype** | | | | | |
|  | Mesenchymal | 90 | 30 | 157 | 37 |
|  | Proneural | 93 | 57 | 226 | 83 |
|  | Classical | 108 | 72 | 145 | 99 |
| **CIMP Status** | | | | | |
|  | G-CIMP | NA | 23 | 46 | 11 |
|  | Non- G-CIMP | NA | 136 | 482 | 208 |
|  | NA | 393 | 0 | 0 | 0 |
| **IDH1 Status** | | | | | |
|  | Wild-Type | 291 | 98 | 372 | NA |
|  | Mutant | 92 | 35 | 30 | NA |
|  | NA | 0 | 34 | 136 | NA |
| **Gender** | | | | | |
|  | Male | 239 | 108 | 314 | NA |
|  | Female | 154 | 51 | 203 | NA |
| **Age** | | | | | |
|  | <60 | 305 | 98 | 267 | NA |
|  | >60 | 88 | 61 | 252 | NA |

**Table S7.**
